# Nanoscale Diblock Copolymer Micelles: Characterizations and Estimation of the Effective Diffusion Coefficients of Biomolecules Release through Cylindrical Diffusion Model

**DOI:** 10.1371/journal.pone.0105234

**Published:** 2014-08-18

**Authors:** M. Wahab Amjad, Mohd Cairul I. Mohd Amin, Shalela M. Mahali, Haliza Katas, Ismanizan Ismail, M. Naeem ul Hassan, Victor T. Giam Chuang

**Affiliations:** 1 Center for Drug Delivery Research, Faculty of Pharmacy, Universiti Kebangsaan Malaysia, Kuala Lumpur, Federal Territory, Malaysia; 2 School of Informatics & Applied Mathematics, Universiti Malaysia Terengganu, Kuala Terengganu, Terengganu, Malaysia; 3 Institute of Systems Biology, Universiti Kebangsaan Malaysia, Bangi, Selangor, Malaysia; 4 School of Pharmacy, Curtin Health Innovation Research Institute, Faculty of Health Sciences, Curtin University, Perth, Western Australia, Australia; China University of Mining and Technology, China

## Abstract

Biomolecules have been widely investigated as potential therapeutics for various diseases. However their use is limited due to rapid degradation and poor cellular uptake *in vitro* and *in vivo*. To address this issue, we synthesized a new nano-carrier system comprising of cholic acid-polyethylenimine (CA-PEI) copolymer micelles, *via* carbodiimide-mediated coupling for the efficient delivery of small interfering ribonucleic acid (siRNA) and bovine serum albumin (BSA) as model protein. The mean particle size of siRNA- or BSA-loaded CA-PEI micelles ranged from 100–150 nm, with zeta potentials of +3-+11 mV, respectively. Atomic force, transmission electron and field emission scanning electron microscopy demonstrated that the micelles exhibited excellent spherical morphology. No significant morphology or size changes were observed in the CA-PEI micelles after siRNA and BSA loading. CA-PEI micelles exhibited sustained release profile, the effective diffusion coefficients were successfully estimated using a mathematically-derived cylindrical diffusion model and the release data of siRNA and BSA closely fitted into this model. High siRNA and BSA binding and loading efficiencies (95% and 70%, respectively) were observed for CA-PEI micelles. Stability studies demonstrated that siRNA and BSA integrity was maintained after loading and release. The CA-PEI micelles were non cytotoxic to V79 and DLD-1 cells, as shown by alamarBlue and LIVE/DEAD cell viability assays. RT-PCR study revealed that siRNA-loaded CA-PEI micelles suppressed the mRNA for ABCB1 gene. These results revealed the promising potential of CA-PEI micelles as a stable, safe, and versatile nano-carrier for siRNA and the model protein delivery.

## Introduction

Devices and vehicles for drug delivery have made excellent contributions to the improvement of therapeutic outcomes by enhancing the efficacy of established and emerging drugs [1]–[4]. One major milestone in the field of nanomedicine is the development of advanced carriers capable of delivering therapeutic payloads in significant quantities to specific sites [5], [6]. Much of the research in this area has focused on particle-based technologies, such as liposomes, micelles, and nanoparticles [7]–[9].

Polymeric micelles are nano-sized with a core-shell structure, including a hydrophobic core and a hydrophilic shell [10], [11]. The hydrophobic core of micelles may be used as a cargo space for the encapsulation of a variety of hydrophobic therapeutic and diagnostic agents. Such encapsulation substantially increases their bioavailability and improves their pharmacokinetics and biodistribution. The size of micelles permits their extravasation and accumulation in a variety of pathological sites where the permeability of the vascular endothelium is increased, such as infarct zones and tumors. This fact provides a unique opportunity for physiology-based targeting of drugs and/or drug-loaded pharmaceutical carriers, such as micelles, to these pathological areas via the enhanced permeation and retention (EPR) effect [12], [13]. Micelles are also easy to prepare on a large scale, providing an additional practical advantage.

Some endogenous peptides, proteins, and oligonucleotides have attracted significant attention because of their great potential for treating chronic diseases [14]. However, the *in vivo* environment of the human body has tended to limit their therapeutic application [15]. BSA was chosen as a representative protein molecule because of its ligand-binding properties and its practical advantages of being readily available and inexpensive [16]–[18]. Moreover, BSA shares 76% protein sequence homology with human serum albumin (HSA), indicating that the results of the studies conducted here may also be applicable to HSA [19].

Recent results from phase I and phase II clinical studies of siRNAs for age-related macular degeneration (AMD) and respiratory syncytial virus (RSV) infection have demonstrated their therapeutic potential [20]–[22]. Despite the great potential of these rapid advances, the application of RNAi-based therapy to humans in a clinical setting is significantly limited by the short serum half-life and poor cellular uptake of these molecules [23].

To explore the potential of cholic acid-polyethylenimine (CA-PEI) copolymer micelles, we investigated their morphology using a range of different techniques. We also investigated their versatility as carriers of protein (bovine serum albumin, BSA) and oligonucleotides (small interfering ribonucleic acid, siRNA).

## Materials and Methods

### Chemicals and Reagents

CA, PEI (average molecular weight [MW] approximately 1300 g/mol), N,N'-Dicyclohexylcarbodiimide (DCC), N-Hydroxysuccinimide (NHS) and BSA (66 kDa) were purchased from Sigma-Aldrich (St. Louis, Missouri, USA). PureLink RNA Mini Kit, UltraPure DEPC treated (RNase/DNase-free) water, Lipofectamine RNAiMAX, PureLink DNase, RNase*Zap* Solution, SuperScript VILO MasterMix First Strand Synthesis SuperMix for qRT-PCR, dNTP Mix, Taq DNA Polymerase PCR Buffer, PlatinumTaq DNA Polymerase, *Silencer* Select Negative Control No. 1 siRNA, siRNA targeting the ATP-binding cassette sub-family B member 1 (ABCB1) gene (sense sequence: 5′-GUUUGUCUACAGUUCGUAAtt-3′) were purchased from Life Technologies (Carlsbad, California, USA). This human gene encodes P-glycoprotein (P-gp) which is an important cell membrane protein involved in the removal of many foreign substances from cells. Some cancer cells also express large amounts of P-gp, which renders these cancers multi-drug resistant.

### Cell Culture

The human colorectal adenocarcinoma (DLD-1) and Chinese hamster lung fibroblast (V79) cell lines were obtained from the American Type Culture Collection (Manassas, VA, USA). DLD-1 cells were cultured and maintained in RPMI-1640 medium, whereas V79 cells were cultured in DMEM. Both cell lines were supplemented with 10% FBS and 1% penicillin-streptomycin, and were maintained at 37°C in a humidified 5% CO_2_∶95% air atmosphere.

### Synthesis of CA-PEI polymer

**Figure 1:**
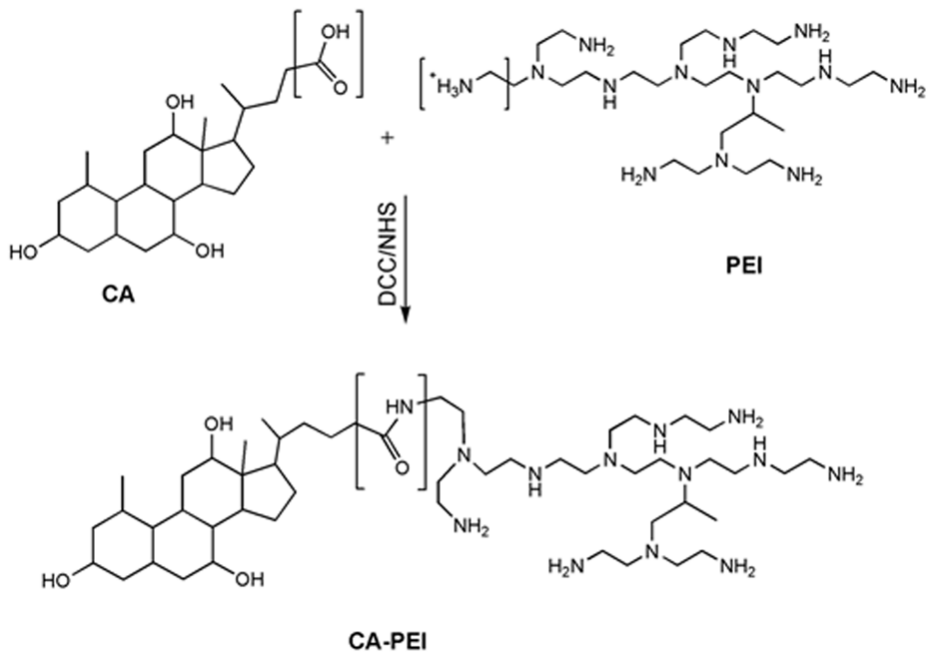
Schematic representation of the synthesis of cholic acid-polyethylenimine (CA-PEI) copolymer via carbodiimide-mediated coupling.

CA-PEI was synthesized *via* carbodiimide mediated coupling (Figure 1). Firstly, CA was activated with DCC and NHS at 25°C for 8 h. The activated CA was then conjugated to the primary amine group of PEI by incubating for 15 h in dichloromethane at different molar ratios (1∶1, 1∶3, 3∶1) of CA to PEI. The resulting conjugates were dried in a rotary evaporator and dissolved in dilute HCl, followed by precipitation with cold acetone. Lastly, they were suspended in deionized water, filtered, and freeze-dried. The mean particle diameter and zeta potential (surface charge) of freshly prepared CA-PEI micelles were determined by dynamic light scattering using ZS-90 Zetasizer (Malvern Instruments, Worcestershire, UK).

### Transmission electron microscopy (TEM)

Micelle morphology was investigated by transmission electron microscopy (TEM; FEI Tecnai Spirit, Eindhoven, Netherlands) at an accelerating voltage of 120 kV. A small drop of each aqueous copolymer solution was deposited onto a copper grid coated with carbon film. Excess copolymer solution was wiped off using filter paper, and the grid was dried under ambient atmosphere for 1 h. The specimen on the copper grid was negatively stained with phosphotungstic acid.

### Atomic force microscopy (AFM)

The morphology of the CA-PEI micelles was analyzed by atomic force microscope NTEGRA Prima (NT-MDT, Russia). The scanner (50 µm) equipped with capacitive sensors and silicon cantilevers (curvature 10 nm, spring constant 0.4–2.7 N/m, NT-MDT) was used for scanning. An aliquot of the suspension of CA-PEI was evenly spread on the surface of freshly-cleaved mica and dried. The samples were analyzed in air using the tapping mode with a resonance frequency of 80 kHz, scan rate of 1 Hz and resolution of 256×256 pixels. The tip loading force was minimized to reduce deformation of the sample.

### Field emission scanning electron microscopy (FESEM)

The morphology of the CA-PEI micelles was also examined by FESEM (JEOL JSM-6700F) at an accelerating voltage of 10 kV. Samples were prepared in liquid nitrogen, followed by platinum coating using a JEOL JFC-1100E ion-sputtering device.

### BSA Loading

BSA solution was prepared in phosphate buffered saline (PBS). CA-PEI copolymers with different molar ratios were dissolved in methanol. The BSA solution was then added drop wise to the CA-PEI micelles. Each solution was then mixed with deionized water under ultrasonic agitation using a sonifier (Branson Ultrasonics Co., Danbury, CT, USA) at a power level of 3 for 20 s. The organic solvents were then completely removed under vacuum using a rotary evaporator. BSA-loaded CA-PEI micelles were separated from the solution by ultracentrifugation (Optima L-100 XP ultracentrifuge with NV 70.1 rotor, Beckman-Coulter, USA) at 14000 rpm for 30 min. The resulting supernatants were decanted and BSA content was analyzed in triplicate using the Bradford protein assay. Assay signals were measured using a UV-vis spectrophotometer at a wavelength of 595 nm (U.V-1601, Shimadzu, Japan).

The percentage BSA loading efficiency (LE) was calculated using the following equation:
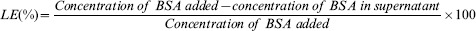
(1)


### siRNA loading

siRNA was complexed with CA-PEI micelles. Three molar ratios of CA-PEI copolymer were reconstituted in PBS to form micelles. siRNA (40 µg) was then added to each micelle solution. The mixture was vortexed for 15 s and then kept still at room temperature for 30 min in the dark to allow complexation between siRNA and micelles. siRNA LE (%) for CA-PEI micelles was calculated after determination of the free siRNA concentration in the supernatant following centrifugation (35,000 × *g*, 15 min). siRNA concentration was determined using a UV-vis spectrophotometer (Shimadzu 1800) at a wavelength of 260 nm. Supernatants collected from empty CA-PEI micelles were used as a reference. siRNA LE (%) was calculated using the following equation (Table 1):
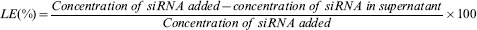
(2)


**Table 1:** Zeta potentials and LE (%) of CA-PEI micelles.

micelles	zeta potential (mV, n = 3)			LE (%, n = 3)		
	1∶1	1∶3	3∶1	1∶1	1∶3	3∶1
CA-PEI	+12.2±1.2*	+18.7±1.5*	+9.2±1.1*			
siRNA-loaded CA-PEI	+6.8±0.7	+3.2±0.2^**^	+7.8±1.0	95±0.72*	97±0.94* ^∞^	93.7±0.85^∞^
BSA-loaded CA-PEI	+10.1±1.1	+11.7±1.3	+8.3±0.8*	70.3±1.6*	74±0.98* ^α^	68±1.3^ α^

*^, **, ∞, *∞, α, *α^ p< 0.05 for the comparison between group means.

### Binding efficiency of siRNA

The binding efficiency of siRNA was investigated by adding 20 µL of siRNA-loaded CA-PEI micelles into the wells of a 4% (w/v) agarose gel with SYBR Green (Invitrogen, Carlsbad, CA, USA). Free siRNA was used as positive control, while the blank CA-PEI micelles were used as negative controls. Electrophoresis was run for 26 min, in accordance with the supplier's protocol (2005-2006, Invitrogen). The siRNA bands were then visualized using a real-time UV transilluminator (Invitrogen).

### *In vitro* release study of BSA and siRNA

BSA and siRNA release experiments were carried out *in vitro* at pH 5 and pH 7.4. CA-PEI micelles with a molar ratio 1∶3 were selected for the studies, as they had the optimal characteristics (small nanosize, net positive charge, high loading and binding efficiency). The BSA- and siRNA-loaded CA-PEI micelles were introduced into dialysis membranes. These were then placed in 100 mL of release medium at either pH 5 or pH 7.4. The beakers were placed on magnetic stirrers with a stirring speed of 100 rpm at 37°C. At suitable intervals, 3 mL samples were taken from the release medium and an equivalent volume of fresh medium was added. The concentration of BSA in each sample was analyzed using the Bradford protein assay and the signals were read by a UV-vis spectrophotometer at 595 nm (U.V-1601, Shimadzu), whereas the amount of released siRNA was analyzed at a wavelength of 260 nm.

### Release kinetics models

To investigate the mechanism of siRNA/BSA release from CA-PEI micelles, various kinetic equations were fitted to the release data and according to the correlation coefficient, zero-order (3), first-order (4), Korsmeyer-Peppas (5), and Higuchi (6) models were chosen to predict the siRNA/BSA release rate, diffusion behavior from CA-PEI micelles, and to understand the physics of siRNA/BSA transport. 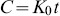
(3)

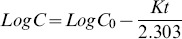
(4)

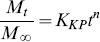
(5)

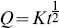
(6)


In these equations, K_0_ was the zero-order rate constant, expressed in units of concentration/time, t was the time in hours, C_0_ was the initial concentration of drug, K was the first-order constant, M_t_/M_∞_ was the fraction of drug released at time t, K_KP_ was the rate constant, n is the release exponent, and k is the constant reflecting the design variables of the system.

### Cylindrical diffusion model

The effective diffusion coefficients of each release experiment were estimated using a cylindrical diffusion model. The dialysis membrane, containing 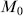 of BSA- or siRNA-loaded CA-PEI, was considered as a cylindrical device with radius 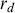 and height 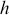. This cylindrical device was placed in a cylindrical container of radius 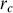 and height 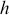, filled with release medium (pH 5 and pH 7.4). Assuming that the diffusion process was radial, the experiment was represented by the following diffusion equation in polar coordinates:
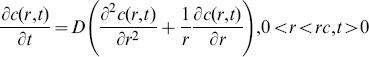
(7a)

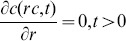
(7b)

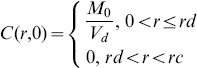
(7c)


Where 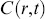 was the concentration at radius *r* and time *t*, and 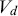 was the volume of the device (the dialysis membrane). In order to model the initial burst release, the effective diffusion coefficient, *D*, was assumed to be a piecewise constant:
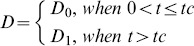



Where *t_c_* was the time at the end of the initial burst release phase. The effective diffusion coefficients *D*_0_, *D*_1_, and the threshold time *t_c_*, were determined using the release experiment data.

The diffusion system (7a)–(7b) could be analytically solved for the concentration 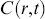. The cumulative release of siRNA- and BSA-loaded CA-PEI (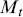) was found by integrating the concentration over the region outside the dialysis membrane.^29^ This value could be summarized as:
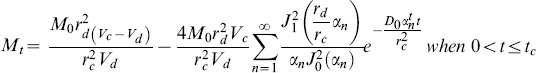
(8a)


and
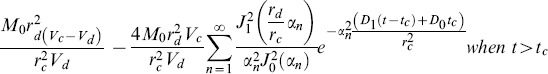
(8b)


Where *V_c_* was the volume of the release medium in the container, *J*_1_ was the first-order Bessel function and 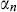 was the root for 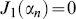 for 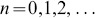. The fraction of the cumulative release, *M_t_*, over the initial loading, *M*_0_, was found by dividing (8a)–(8b) by *M*_0_. Upon substituting the volume of the cylinders with 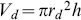 and 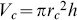 into the resulting equations, the formulae were then simplified to:
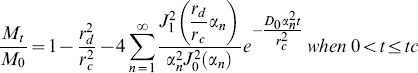
(9a)


and
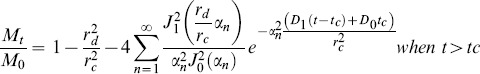
(9b)


The unknown parameters, *D*_0_, *D*_1_, and *t_c_* were then determined by fitting (9a)–(9b) to the experimental release data using a weighted nonlinear least squares method. The aim was to minimize the fitting error. 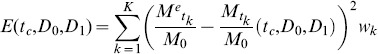



Where 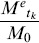
was the experimental value at time *t_k_*, 
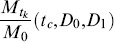
was the value from the formulae at time *t_k_*,when the parameters *t_c_*,*D*_0_, and *D*_1_ were used, and 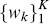
were positive weights. In this study, the infinite series of (9a)–(9b) was truncated for the first 62 terms to evaluate 
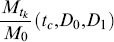
. The values for the non-negative roots of the first-order Bessel function used in the computation were:
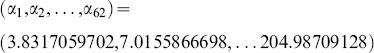



The weights chosen were proportional to the interval between two data points



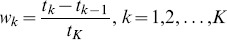



These computation procedures were performed using MATLAB.

### SDS-PAGE

The integrity of micelle BSA following processing and release was analyzed using SDS-PAGE (12% resolving gel and 10% stacking gel) on a Mini Protean System (Bio-Rad, USA). Protein marker, BSA and BSA-loaded CA-PEI micelles were mixed with Laemmli sample buffer in a 1∶1 ratio and heated at 95°C for 5 min. Samples (15 µL) were loaded into the wells and the gel was run using a Mini-Protean System Tetra cell at a constant voltage of 150 V for 90 min with a running buffer containing 25 mM Tris, 192 mM glycine, and 0.1% SDS, at pH 8.3. The sample bands were stained for 40 min with 0.1% Coomassie blue solution containing 40% acetic acid and 10% methanol, followed by destaining overnight with a solution of 40% acetic acid and 10% methanol.

### Circular dichroism (CD)

The secondary structure of BSA released from the CA-PEI micelles following processing was investigated using circular dichroism (CD) spectropolarimetry (Jasco J-810 spectropolarimeter). BSA solution (0.01% in PBS) was used as the control. BSA released from the different molar ratio combinations of BSA-loaded CA-PEI micelles was quantified using the Bradford protein assay, assay signals were read in a UV-vis spectrophotometer at 595 nm (U.V-1601, Shimadzu), and the BSA concentration was adjusted to 0.01%. The CD analysis was performed using a 0.1 cm path length quartz cell and parameters were as follows: 1 nm bandwidth, 10 mdeg sensitivity, 1 nm resolution, 4 s response time, and 100 nm min^−1^ scanning. The samples were scanned over a far-UV range of 200–240 nm for secondary structure analysis. The secondary structures (α-helix and β-strand) of control and released BSA were estimated using K2D3 software.

### Serum stability study of siRNA

The siRNA-loaded CA-PEI micelles were subjected to stability testing in serum. The siRNA loaded CA-PEI micelles were incubated at 37°C with an equal volume of Dulbecco's Modified Eagle's Medium (DMEM) supplemented with 5% fetal bovine serum (FBS) and 1% penicillin-streptomycin. Free siRNA given the same treatment acted as a control in the experiment. At each time interval (0 and 30 min, 1, 2, 4, 7, 24, and 48 h), 50 µL of the mixture was removed and stored at −20°C until gel electrophoresis was performed. Prior to gel electrophoresis, samples were incubated in a bath incubator for 5 min at 80°C to terminate serum activity. Then, 5 µL heparin (1000 U/mL) was added to displace siRNA from the micelles. The integrity of the complexes was analyzed by gel electrophoresis using 4% (w/v) agarose gel with SYBR Green (Invitrogen). Electrophoresis was run for 26 min in accordance with the manufacturer's protocol (2005–2006, Invitrogen). The siRNA bands were then visualized under a real-time UV transilluminator (Invitrogen).

### In vitro cytotoxicity assay

The seeded cells (DLD-1 and V79) in the 96-well cell culture plates were treated with BSA- and siRNA-loaded CA-PEI micelles. After 24 h and 48 h incubations with BSA- and siRNA-loaded CA-PEI micelles at 37°C, a final dilution of 1/10 per cell volume of alamarBlue reagent was added to the treated cells, followed by a 4-h incubation prior to analysis. The absorbance of each sample at 570 nm (A570) was measured using a microplate reader (Varioskan Flash, Thermo Scientific, Waltham, MA, USA).

### LIVE/DEAD cell viability assay

The LIVE/DEAD cell viability assay was performed using 96 well plates. Briefly, 30,000 cells were seeded in each well and treated with BSA- or siRNA-loaded micelles for 24 or 48 h. Subsequently, the cells were rinsed twice with PBS before the fluorochromes were added and incubated for 45 min. The reagents were then removed and the cells were analyzed for multicolor fluorescence using a Floid Cell Imaging Station (Molecular Probes Life Technology, France) for calcein and ethidium homodimer.

### Investigation of ABCB1 mRNA suppression using RT-PCR

The total RNA from DLD-1 cells was isolated and purified using PureLink RNA Mini Kit and PureLink DNase as recommended by the manufacturer. The RNA was quantified using Infinite 200 PRO NanoQuant (Tecan, Switzerland). cDNA was prepared from 1.5 µg total RNA by reverse transcription using SuperScript VILO MasterMix for RT-qPCR according to the manufacturer's instructions. cDNA was also quantified using Infinite 200 PRO NanoQuant. The mastermix for rtPCR was prepared by using nuclease free water, dNTP, MgSO_4_, PCR buffer and PlatinumTaq DNA Polymerase. For ABCB1 gene, the forward and reverse primers were 5′ CGAGGTCGGAATGGATCTTG 3′ and 5′ GCCATTCTGAAACACCACT 3′, whereas for reference gene GAPDH (Glyceraldehyde 3-phosphate dehydrogenase) the forward and reverse primers were 5′ ACCACAGTCCATGCCATCAC 3′ and 5′ TCCACCACCCTGTTGCTGTA 3′, respectively. The RT-PCR was carried out in Eppendorf Mastercycler Nexus Gradient Thermal Cycler (Hamburg, Germany). Amplification products were resolved by 1% agarose gel electrophoresis at 90 V for 60 min in TAE (Tris-acetate-EDTA) buffer and visualized by ethidium bromide staining. The images of the gel were taken by an image reader (Fujifilm LAS-3000, Tokyo, Japan). The densitometric analysis was performed using ImageJ software.

### Statistical Analysis

All data are expressed as mean ± SD of at least three measurements. Statistical analysis of all data was performed using one-way analyses of variance (one-way ANOVA), followed by the post hoc Tukey test for multiple comparisons using SPSS for Windows v.19, except for RT-PCR (where post hoc Dunnett test was applied after one-way ANOVA). A p value of <0.05 was considered statistically significant. For the cylindrical diffusion model, the data for the D0 and D1 has been provided in supplementary information.

## Results and Discussion

The cholic acid (CA) was linked to polyethylenimine (PEI) by carbodiimide mediated coupling (Figure 1). Micelles were formed by extensive vortexing of the CA-PEI copolymer in an aqueous buffer. CA-PEI conjugate critical micelle concentration (CMC) values were low (in the high nanomolar to low micromolar range). Functional group characterization was performed by fourier transform infrared spectroscopy, which confirmed the presence of an amide linkage between CA and PEI. Further characterization of the CA-PEI was also performed using nuclear magnetic resonance (NMR) spectroscopy (BrukerAvance III, FT-NMR 600 MHz with cryoprobe, Germany). The average particle size of all molar ratios of blank CA-PEI micelles ranged from 100-150 nm. The micellar surface charge was positive for all the molar ratios tested. The CA-PEI micelles with the highest PEI content (i.e. 1∶3 molar ratio), had the highest positive zeta potential (+18.7±1.5) and vice versa (Table 1).

**Figure 2:**
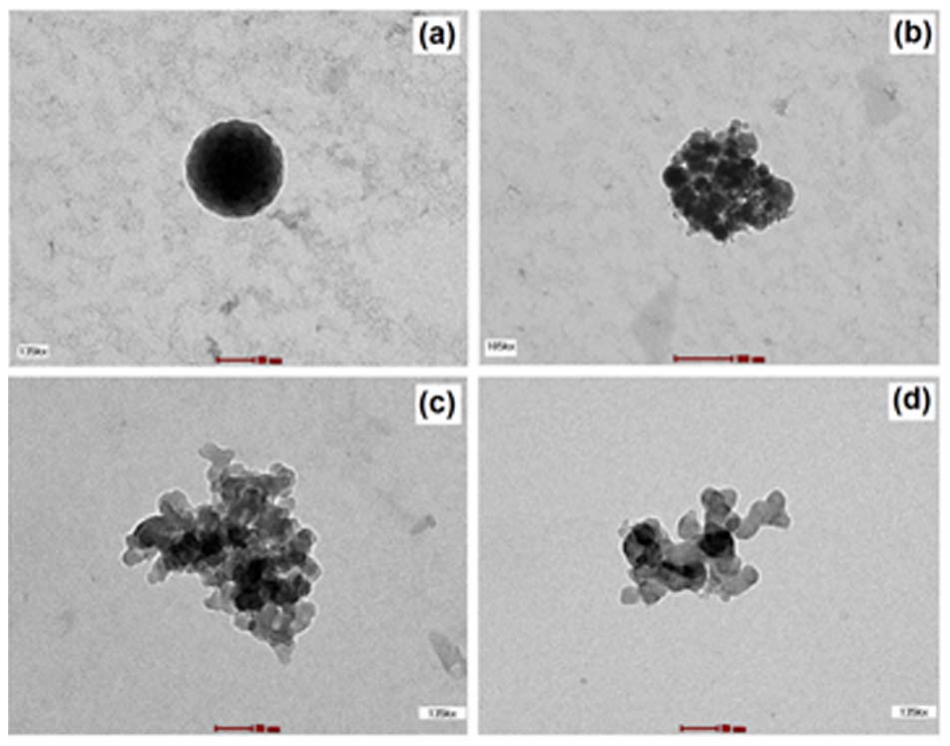
Transmission electron microscope (TEM) images of (a) blank CA-PEI micelles (135 kx); (b) aggregates of blank CA-PEI micelles (105 kx); (c) aggregates of BSA-loaded CA-PEI micelles (135 kx); (d) aggregates of siRNA-loaded CA-PEI micelles (135 kx).

**Figure 3:**
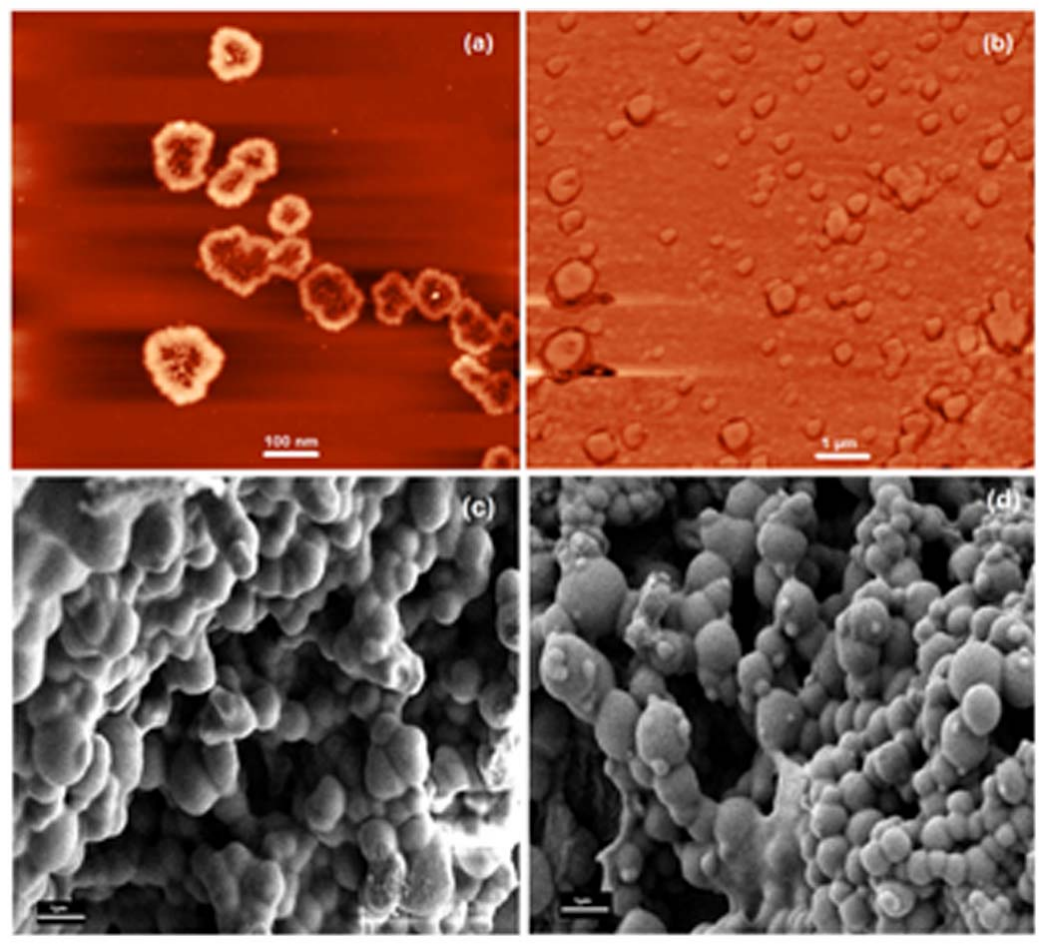
Atomic force microscopy (AFM) and field emission scanning electron microscopy (FESEM) images of CA-PEI micelles. (a) AFM of CA-PEI micelles; (b) AFM of siRNA-loaded CA-PEI micelles; (c) FESEM of BSA-loaded CA-PEI micelles; (d) FESEM of siRNA-loaded CA-PEI micelles. All these images were of micelles with a CA-PEI molar ratio of 1∶3.

Micelle morphology was investigated by transmission electron microscopy (TEM) Figure 2(a)–(d). The atomic force (AFM) (Figure 3(a), (b)) and field emission scanning electron microscopies (Figure 3(c), (d)) of the copolymer micelles were also recorded. The amphiphilic CA-PEI copolymers self-assembled into stable, uniform, spherical micelles in water. Analysis of TEM, AFM and FESEM images also indicated that the micelle diameters at all CA-PEI molar ratios ranged from 100 to 150 nm in aqueous solution (pH 7). The micelles appeared spherical in shape and aggregates were observed.

The percentage of BSA loading efficiency (LE) shown in Table 1 was calculated. The LE (%) values achieved for BSA were influenced by the amount of PEI in the CA-PEI copolymer. Micelles with a CA:PEI molar ratio of 1∶1 and 1∶3 showed higher LE than those with a 3∶1 molar ratio.

siRNA was complexed with CA-PEI micelles. The nitrogen to phosphate ratios (N/P) of siRNA-loaded CA-PEI micelles were 5, 15 and 5 for the molar ratios 1∶1, 1∶3 and 3∶1, respectively. The zeta potential of CA-PEI (1∶3) micelles after siRNA loading decreased to +3.2±0.2. The decrease in the zeta potential was the highest among all the molar ratios. CA-PEI (1∶3) micelles have higher PEI than any other molar ratios, and hence more amine groups are available for interaction. Thus, more siRNA will be interacted with the available amine groups resulting in its neutralization and hence decrease the zeta potential. The LE (%) of siRNA-loaded CA-PEI micelles increased from 93%±0.85 to 97%±0.94 with increasing PEI content, and the difference between siRNA LE at a CA-PEI concentration ratio of 1∶1 and 1∶3 was statistically significant. This indicated that siRNA LE (%) was influenced by the CA-PEI concentration ratio. Little free siRNA was detected, indicating that most siRNA was loaded and formed stable complexes with the micelles. High LE (%) was achieved due to the strong electrostatic attraction between siRNA and the oppositely-charged PEI, which led to the formation of tight complexes that entrapped siRNA firmly in the complexes [24], [25].

**Figure 4:**
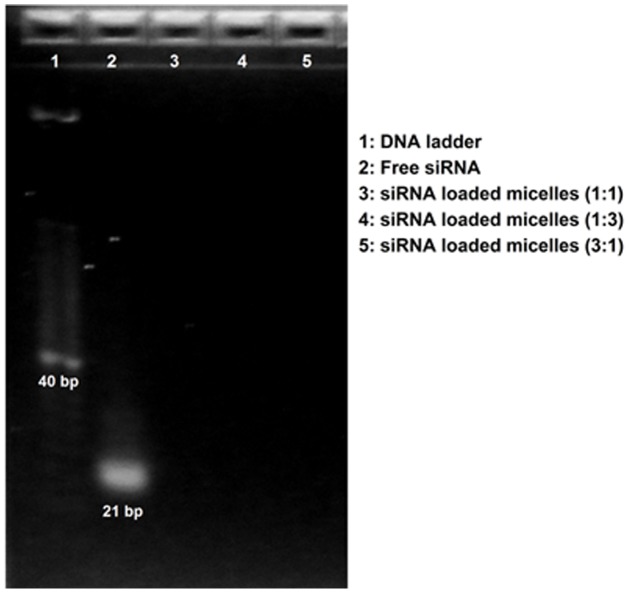
Agarose gel electrophoresis demonstrating the binding efficiency of siRNA complexed with CA-PEI micelles.

The binding efficiency of siRNA was investigated. As shown in Figure 4, it appeared that siRNA bound strongly to the CA-PEI micelles, as no siRNA band was detected in lanes loaded with any of the molar ratio combinations of siRNA-loaded CA-PEI micelles. This absence of bands indicated that no siRNA migration occurred, which reflected the strength of interaction between CA-PEI and siRNA. These findings suggested that upon complexation, negatively charged siRNA phosphate groups interacted with protonated PEI amine groups, binding strongly to form tight complexes [24], [25].

**Figure 5:**
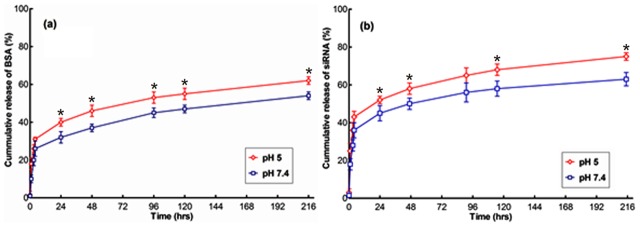
Cumulative *in vitro* release profiles of (a) BSA and (b) siRNA at pH 5 and 7.4. *p< 0.05 for the comparison between group means.

BSA Figure 5(a) and siRNA Figure 5(b) release experiments were carried out *in vitro* at pH 5 and pH 7.4. The first layer of BSA was adsorbed onto the micelle surface purely by virtue of its electrostatic interaction with the CA-PEI micelles. The adsorption of subsequent layers of BSA was mainly governed by intermolecular hydrophobic interactions, because electrostatic interactions were reduced as the surface BSA thickness increased. For this reason, a number of the BSA layers adsorbed onto the CA-PEI micelle surface were not tightly bound [26]. The initial burst release of BSA from the micelle surface, shown in Figure 5, was mainly due to desorption of these loosely-bound BSA molecules. The release of BSA increased with decreasing release medium pH. There was an adsorption-desorption equilibrium between the BSA adsorbed on the surface of the nanoparticle and the BSA released in the medium. The later-stage BSA release was mainly controlled by particle dissolution, releasing tightly bound BSA molecules. As the pH of the buffer solution decreased, the H^+^ concentration increased. The increase in H^+^ concentration forced the dissolution equilibrium to be shifted towards the right, facilitating dissolution of micelles. Both micelle dissolution and BSA release occurred more rapidly at pH 5, compared to pH 7.4.

To investigate the mechanism of siRNA/BSA release from CA-PEI micelles, various kinetic equations were fitted to the release data. The release data modeling is summarized in Table 2. The release study also confirmed that siRNA/BSA release data adequately fitted zero-order, first-order, Korsmeyer-Peppas, and Higuchi models with R^2^ values greater than 0.54. However, the Korsmeyer-Peppas model provided the best mathematical description of siRNA/BSA release, with R^2^ values greater than 0.913. To characterize the release mechanism, the Korsmeyer-Peppas release exponent (n) was calculated [27]. These results indicated that n ranged from 0.18 to 0.26, which confirmed that Fickian diffusional release was involved. On the other hand, a higher burst release of siRNA/BSA occurred at pH 5.

**Table 2:** Model fitting for siRNA/BSA release profiles from CA-PEI micelles at pH 5 and 7.4.

siRNA								
pH	zero-order		first-order		Korsmeyer-Peppas		Higuchi	
	K	R^2^	K	R^2^	n	R^2^	K	R^2^
5	0.1976	0.7478	0.0017	0.6161	0.1848	0.9553	3.318	0.9101
7.4	0.1746	0.6913	0.0018	0.5494	0.2111	0.9304	2.987	0.8732
**BSA**								
5	0.1803	0.791	0.002	0.6539	0.2129	0.9745	2.995	0.9398
7.4	0.1732	0.7875	0.0024	0.5776	0.2684	0.9136	2.8602	0.9244

**Figure 6:**
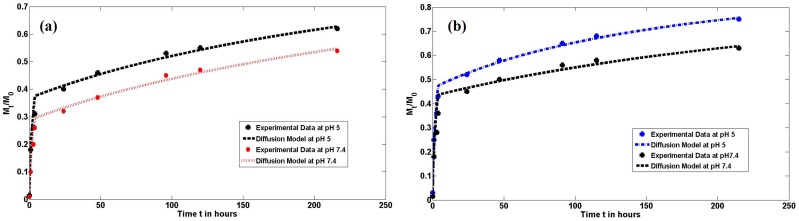
Curves fitted using the cylindrical diffusion model for cumulative *in vitro* release profiles of (a) BSA and (b) siRNA at pH 5 and pH 7.4.

The effective diffusion coefficients of BSA Figure 6(a) and siRNA Figure 6(b) release were estimated using a cylindrical diffusion model [28]. Table 3 lists the computed errors, the effective diffusion coefficients, and the threshold time for siRNA and BSA release experiments in pH 5 and pH 7.4 release media. The diffusion coefficients during the initial burst phase were about 20–30 times larger than the subsequent diffusion coefficients. The diffusion coefficients for the release experiments in pH 5 release medium were 17–54% larger than those in pH 7.4 release medium. All four experiments showed that the optimal threshold time was 4 h.

**Table 3:** The computed errors, the effective diffusion coefficients, and the threshold time for siRNA and BSA release experiments in pH 5 and pH 7.4.

	D_0_(cm^2^/s)	D_1_(cm^2^/s)	t_c_ (h)	Error
**BSA, pH 5**	1.37×10^−6^	6.22×10^−8^	4	1.42×10^−2^
**BSA, pH 7.4**	8.27×10^−7^	4.55×10^−8^	4	1.41×10^−2^
**siRNA, pH 5**	2.31×10^−6^	1.24×10^−7^	4	7.61×10^−4^
**siRNA, pH 7.4**	1.91×10^−6^	5.72×10^−8^	4	2.66×10^−2^

**Figure 7:**
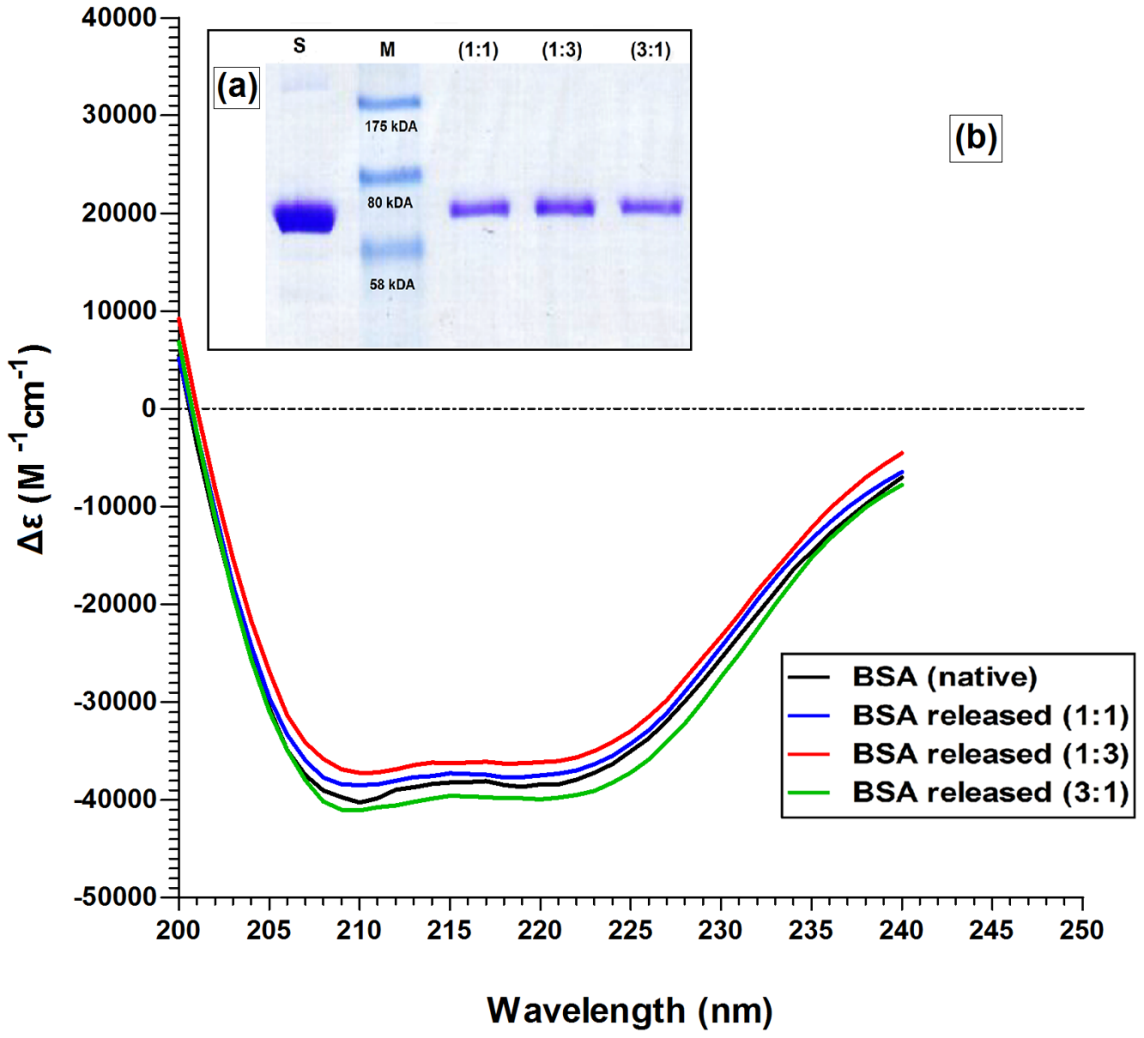
(a) SDS-PAGE and (b) circular dichroism analyses of BSA released from different molar ratio CA-PEI micelles (1∶1, 1∶3, and 3∶1).

The integrity of micelle BSA following processing and release was analyzed using SDS-PAGE (12% resolving gel and 10% stacking gel) Figure 7(a). The bands observed confirmed that BSA that had endured the micelle loading process at 37°C did not differ from freshly prepared BSA standards. Therefore, it could be concluded that BSA remained intact and did not aggregate in the CA-PEI micelles under these experimental conditions. Because SDS-PAGE analysis only provided information relating to the molecular weight of the protein, circular dichroism (CD) was also carried out to provide additional secondary structural information.

The secondary structure of BSA released from the micelles following processing was conformed using CD. Figure 7(b) showed that the CD spectra of BSA released from all three molar ratios of CA-PEI micelles were identical to that of the BSA standard solution. These CD spectra exhibited the two characteristic negative bands of α-helical structure of protein at 208 nm and 222 nm. The secondary structure (α-helix and β-strand) of BSA was estimated by using K2D3 software. The α-helix contents of the releases and standard BSA were found between 65–68% (Table 4). These findings suggest that the secondary structure of BSA was preserved throughout micelle loading and release processes.

**Table 4:** The amount of secondary structure (α-helix and β-strand) of BSA was estimated by using K2D3 software.

Sample	α-helix (%)	β-strand (%)
BSA (Standard)	66.63±2.41	7.69±1.11
CA-PEI (1∶1)	66.23±1.95	7.39±1.19
CA-PEI (1∶3)	66.33±2.17	7.45±1.31
CA-PEI (3∶1)	66.07±2.12	7.23±1.11

**Figure 8:**
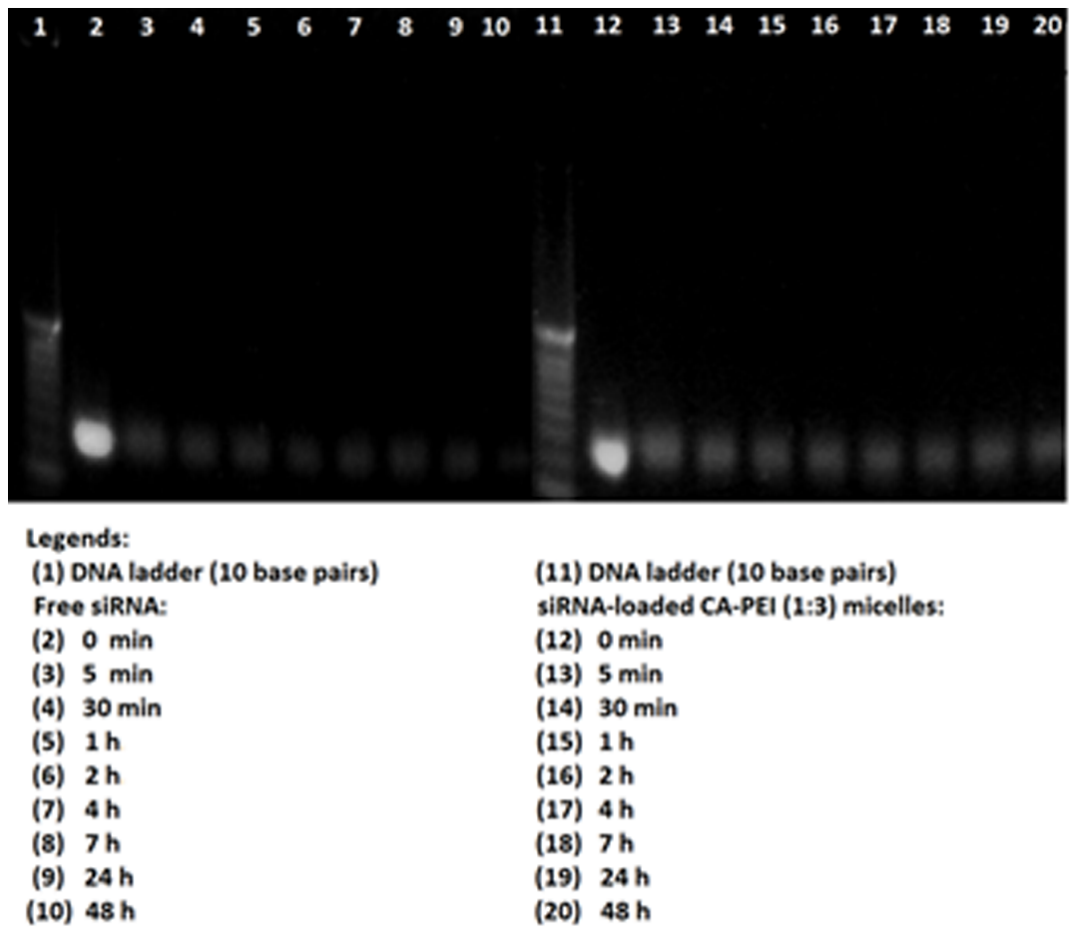
Agarose gel electrophoresis to determine the stability of siRNA-loaded CA-PEI (1∶3) micelles in FBS.

The siRNA-loaded CA-PEI micelles were subjected to stability testing in fetal bovine serum (FBS) (Figure 8). The figure shows trailing bands for siRNA-loaded CA-PEI (1∶3) micelles, indicating the migration of siRNA from these complexes following heparin treatment. The ability to remain stable in FBS is an important determinant of the siRNA molecule's likelihood of gaining access to cells and exerting biological effects [29]. These analyses found that free siRNA had started to degrade after 5 min incubation in 5% FBS at 37°C, with most of it degraded after 48 h incubation (lane 10). In contrast, CA-PEI micelles effectively protected siRNA from nuclease degradation, even after 48 h incubation, as a brighter band of siRNA was seen in lane 20.Hence, it was assumed that siRNA had been partially unbound from the complexes or interacted with components in FBS after being released from the complexes. This study also showed that naked siRNA migrated further than siRNA that had been complexed with micelles. This might be due to nuclease degradation of naked siRNA, resulting in lighter bands that migrated further. Alternatively, CA-PEI micelles may have effectively protected siRNA from nuclease degradation, resulting in heavier bands that migrated shorter distances. Interestingly, previous work indicated that partially degraded siRNA may still retain functional activity, as partial degradation did not always correlate with loss of activity [30]. For maximal activity in cells, siRNA must be stable from nuclease digestion. The results showed that CA-PEI micelles effectively protected the siRNA from nuclease degradation *in vitro*. Thus, it is expected to produce protection from nucleases degradation *in vivo* as well as resulting in gaining access to cells and exert biological effects. The carrier plays an important role to protect its cargo from nuclease degradation for efficient gene delivery. Moreover, the cross linker also plays a vital role in stability of nanoparticles. Thus, siRNA protection can be improved by choosing highly suitable carrier and crosslinker for efficient delivery to target site.

**Figure 9:**
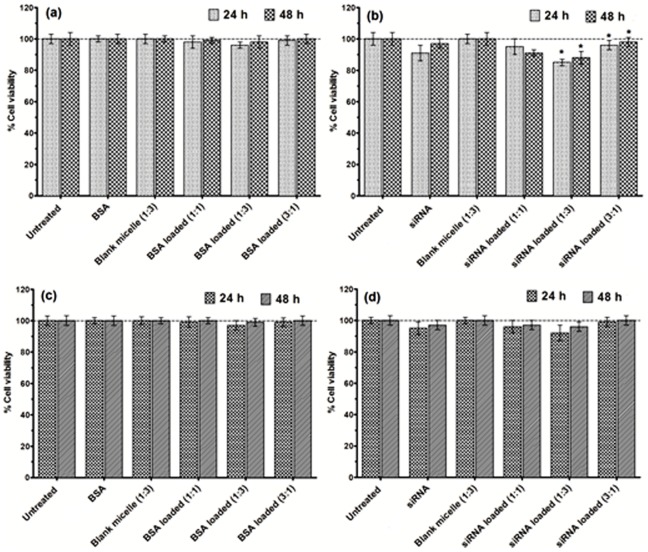
*In vitro* cell viability assays following exposure of V79 (a, b) and DLD-1 (c, d) cells to BSA-loaded CA-PEI micelles (a, c) or siRNA-loaded CA-PEI micelles (b, d). * p<0.05 for the comparison between group means.

The effect of BSA- Figure 9(a), (c) and siRNA-loaded CA-PEI micelles Figure 9(b), (d) on the viability of human colorectal adenocarcinoma (DLD-1) and Chinese hamster lung fibroblast (V79) cell lines was investigated by alamarBlue assay. This study includes siRNA against ABCB1 gene which is responsible for the multi-drug resistance. This multi-drug resistance is a major hindrance in the treatment of various diseases including cancer. The aim behind using both V79 (lung fibroblast, normal) and DLD-1 (colorectal adenocarcinoma, cancer) cell lines was to provide an insight on the effect of siRNA-loaded CA-PEI micelles against normal as well as cancer cells. Moreover, as the CA-PEI micelles are novel, the effect of blank micelles as well as siRNA- and BSA-loaded CA-PEI micelles on normal and cancer cells were carried out in order to investigate any unexpected potentiating cytotoxic effect caused by loading of either siRNA or BSA. No significant reduction in cell viability was observed in either cell line treated with BSA or BSA-loaded CA-PEI micelles at different CA:PEI molar ratios Figure 9(a), (c). Only a slight reduction in cell viability was detected when V79 and DLD-1 cells were treated with free siRNA and siRNA-loaded CA-PEI micelles Figure 9(b), (d). There was a statistically significant reduction in cell viability in V79 cells treated with siRNA-loaded CA-PEI (1∶3), as compared to the untreated cells. This may be due to the higher entrapment efficiency of siRNA in CA-PEI (1∶3) micelles, combined with a higher transfection efficiency associated with the increased PEI content. These findings suggested that CA-PEI micelles were non-toxic and therefore have great potential as vectors for intracellular delivery of siRNA and proteins.

In order to confirm and visualize the above findings, an assay was performed using the LIVE/DEAD Viability/Cytotoxicity Kit for mammalian cells. This assay provided information about the functional status of the cell by detecting cytoplasmic esterase activity and membrane disruption using two fluorescent dyes, calcein AM and ethidium homodimer. Calcein AM crossed the cell membrane. When hydrolyzed by cytoplasmic esterase (live cells), calcein AM fluoresced at a wavelength of 515 nm (green). Ethidium homodimer fluoresced at 617 nm (red) after binding to DNA. Red fluorescence only appeared in cells where the cell membrane was disrupted (dead cells).

**Figure 10:**
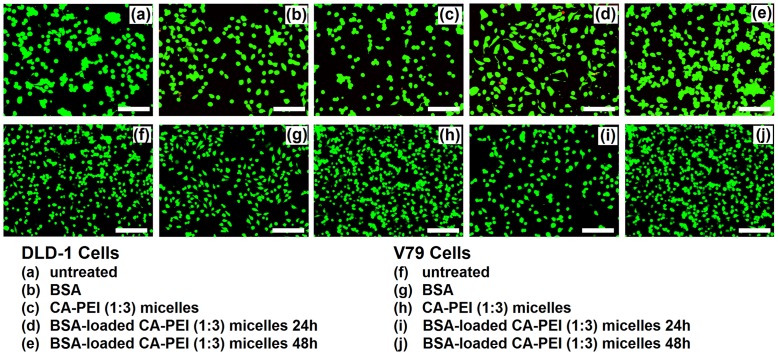
LIVE/DEAD cell viability assay of DLD-1 and V79 cells after treatment with BSA-loaded CA-PEI (1∶3) micelles. Scale bar 20µm

**Figure 11:**
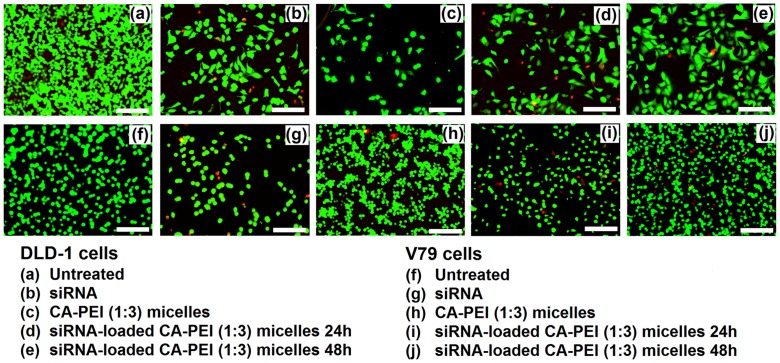
LIVE/DEAD cell viability assay of DLD-1 and V79 cells after treatment with siRNA-loaded CA-PEI (1∶3) micelles. Scale bar 20µm

In agreement with the results of the alamarBlue cell viability assay, no significant reduction in cell viability was observed following exposure to the CA-PEI micelles. Moreover, cells exposed to the BSA-loaded CA-PEI micelles also exhibited more than 95% viability Figure 10(a)–(j). The viability of cells, which were treated with either free siRNA, or siRNA-loaded CA-PEI micelles, was reduced. A significant reduction in V79 cell viability was seen following treatment with siRNA-loaded CA-PEI micelles Figure 11(a)–(j). This reduction was due to the presence of siRNA. This assay complemented the results of the alamarBlue assay.

**Figure 12:**
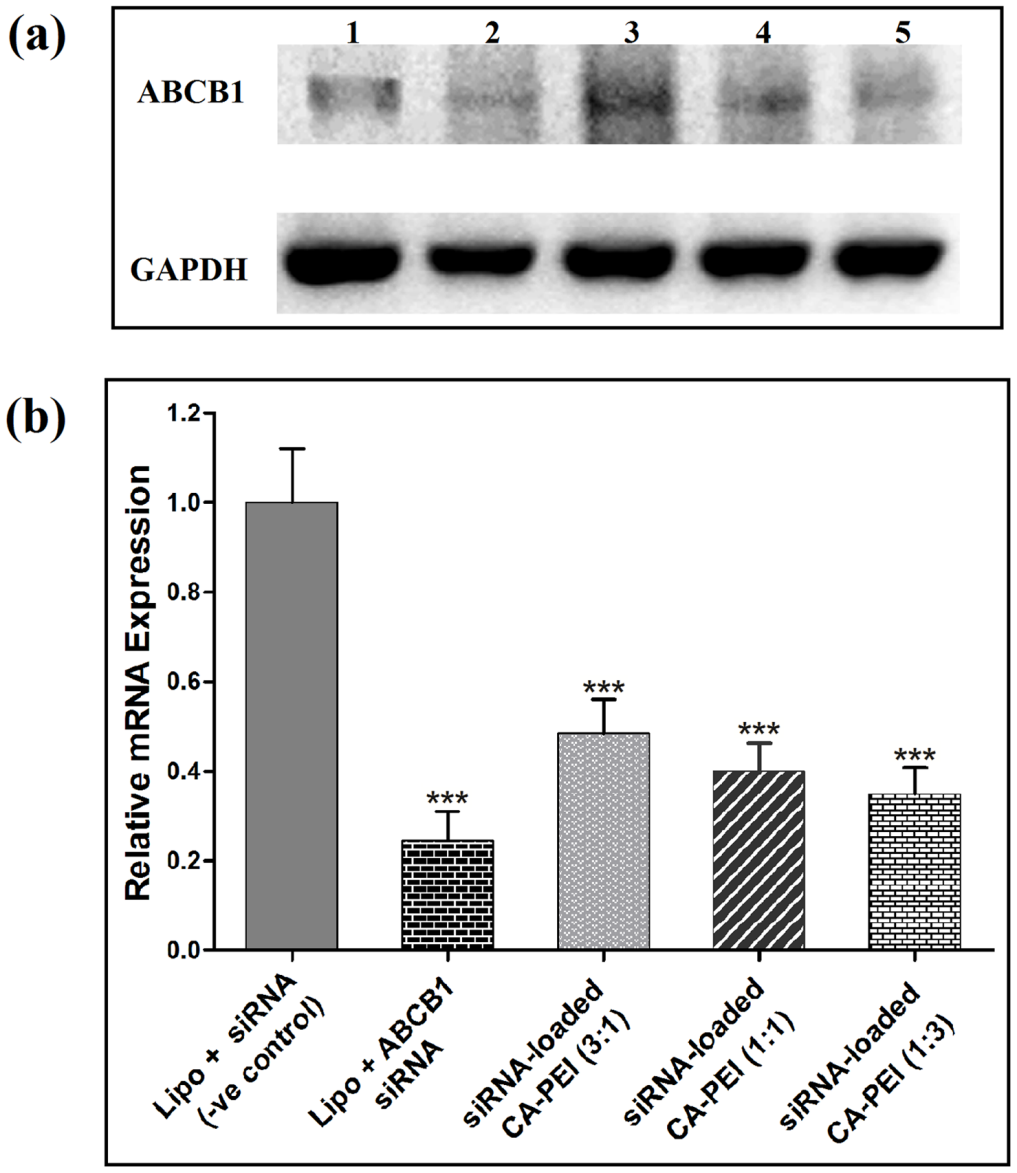
Analysis of ABCB1-mRNA suppression using RT-PCR. 1% agarose gel electrophoresis showing bands of ABCB 1 and GAPDH (a) and graph showing relative mRNA expression in DLD 1 cells after treatment with different samples (b). * p< 0.05 for the comparison between group means.

The primary measure of siRNA-loaded CA-PEI micelles-mediated effects is the silencing of the target mRNA, which should result in the corresponding reduction in ABCB1 gene levels. To test the silencing of ABCB1 mRNA, the RT-PCR method was used in order to determine ABCB1-mRNA in DLD 1 cells by three different molar ratio combinations of CA-PEI micelles loaded with siRNA. The concentration of siRNA was kept constant in all three molar ratio combinations. As shown in Figure 12(a), ABCB1-mRNA levels were down regulated in DLD-1 cells transfected with siRNA-loaded CA-PEI micelles after 24 h. A silencing effect was found to be negligible for the negative control siRNA. The mRNA silencing effect of siRNA-loaded CA-PEI (1∶3) micelles was the highest Figure 12(b) as it had greater content of PEI which itself has exhibited noteworthy cell transfection potential in the past. This finding may indicate that mRNA silencing is clearly relevant to the ability of siRNA to transfect the cells. The combination of Lipofectamine and siRNA exhibited the highest level of mRNA silencing. Interestingly, the silencing efficiency of siRNA delivered with CA-PEI (1∶3) was comparable to that of lipofectamine, indicating potential usefulness of CA-PEI micelles in non-viral siRNA delivery.

## Conclusion

We have shown that micelles made from CA-PEI conjugates can be effectively loaded with siRNA and BSA. The integrity of siRNA and BSA loading onto CA-PEI micelles was intact after processing, release, and following incubation with serum. The effective diffusion coefficients of siRNA and BSA release were successfully estimated using a mathematically-derived diffusion model and the release data of siRNA and BSA closely fitted into this model. The CA-PEI micelles were non cytotoxic to V79 and DLD-1 cells. Moreover, the siRNA-loaded CA-PEI micelles were found to be effective in suppressing mRNA of ABCB1 gene. These findings indicate the significant therapeutic potential of CA-PEI micelles as a nano-carrier for oligonucleotide and protein delivery.

## Supporting Information

Table S1 **Statistical analysis of BSA release from CA-PEI micelles.**
(PDF)

Table S2 **Statistical analysis of siRNA release from CA-PEI micelles.**
(PDF)

Table S3 **Estimated values of D calculated from the cylindrical diffusion model.**
(XLS)

Table S4 **Statistical analysis of the effect of BSA- and siRNA-loaded CA-PEI micelles on the cell viability of DLD-1 cells.**
(PDF)

Table S5 **Statistical analysis of the effect of BSA- and siRNA-loaded CA-PEI micelles on the cell viability of V79 cells.**
(PDF)

Table S6 **Statistical analysis of the BSA and siRNA loading efficiency of CA-PEI micelles.**
(PDF)

Table S7 **Statistical analysis of the zeta potential of blank, BSA- and siRNA-loaded CA-PEI micelles.**
(PDF)
