# Supplementary material for: Nanoscale Diblock Copolymer Micelles: Characterizations and Estimation of the Effective Diffusion Coefficients of Biomolecules Release through Cylindrical Diffusion Model
Source: PLoS One. 2014 Aug 18;9(8):e105234. doi: 10.1371/journal.pone.0105234 (PMC4136833; doi:10.1371/journal.pone.0105234)
Supplement: Table S1 — Statistical analysis of BSA release from CA-PEI micelles. (PDF) [file pone.0105234.s001.pdf]

```
NEW FILE.
DATASET NAME DataSet2 WINDOW=FRONT.
T-TEST GROUPS=Formulation(1 2)
  /MISSING=ANALYSIS
  /VARIABLES=Release
  /CRITERIA=CI(.95).
```

T-Test 0h

Notes

Output Created		12-JUL-2014 21:30:45
Comments		
Input	Active Dataset	DataSet2
	Filter	<none>
	Weight	<none>
	Split File	<none>
	N of Rows in Working Data File	6
Missing Value Handling	Definition of Missing	User defined missing values are treated as missing.
	Cases Used	Statistics for each analysis are based on the cases with no missing or out-of-range data for any variable in the analysis.
Syntax		T-TEST GROUPS=Formulation(1 2) /MISSING=ANALYSIS /VARIABLES=Release /CRITERIA=CI(.95).
Resources	Processor Time	00:00:00.00
	Elapsed Time	00:00:00.00

```
[DataSet2] 0 time
```

Group Statistics

	Formulation	N	Mean	Std. Deviation	Std. Error Mean
Release	BSA ph5	3	1.5000	.62450	.36056
	BSA ph7.4	3	1.0667	.60277	.34801

Independent Samples Test

		Levene's Test for Equality of Variances		t-test for Equality of Means	
		F	Sig.	t	df
Release	Equal variances assumed	.037	.856	.865	4
	Equal variances not assumed			.865	3.995

Independent Samples Test

		t-test for Equality of Means			
					95% Confidence ...
		Sig. (2-tailed)	Mean Difference	Std. Error Difference	Lower
Release	Equal variances assumed	.436	.43333	.50111	-.95797
	Equal variances not assumed	.436	.43333	.50111	-.95866

Independent Samples Test

		t-test for Equality of Means
		95% Confidence Interval of the ...
		Upper
Release	Equal variances assumed	1.82464
	Equal variances not assumed	1.82533

```
T-TEST GROUPS=Formulation(1 2)
 /MISSING=ANALYSIS
 /VARIABLES=Release
 /CRITERIA=CI(.95).
```

T-Test 1h

Notes

Output Created		12-JUL-2014 21:32:52
Comments		
Input	Active Dataset	DataSet2
	Filter	<none>
	Weight	<none>
	Split File	<none>
	N of Rows in Working Data File	6
Missing Value Handling	Definition of Missing	User defined missing values are treated as missing.
	Cases Used	Statistics for each analysis are based on the cases with no missing or out-of-range data for any variable in the analysis.
Syntax		T-TEST GROUPS=Formulation(1 2) /MISSING=ANALYSIS /VARIABLES=Release /CRITERIA=CI(.95).
Resources	Processor Time	00:00:00.00
	Elapsed Time	00:00:00.00

Group Statistics

	Formulation	N	Mean	Std. Deviation	Std. Error Mean
Release	BSA ph5	3	18.0667	3.29292	1.90117
	BSA ph7.4	3	10.0000	2.00000	1.15470

Independent Samples Test

		Levene's Test for Equality of Variances		t-test for Equality of Means	
		F	Sig.	t	df
Release	Equal variances assumed	1.531	.284	3.627	4
	Equal variances not assumed			3.627	3.299

Independent Samples Test

		t-test for Equality of Means			
		Sig. (2-tailed)	Mean Difference	Std. Error Difference	95% Confidence ...
					Lower
Release	Equal variances assumed	.022	8.06667	2.22436	1.89085
	Equal variances not assumed	.031	8.06667	2.22436	1.33705

Independent Samples Test

		t-test for Equality of Means
		95% Confidence Interval of the ...
		Upper
Release	Equal variances assumed	14.24248
	Equal variances not assumed	14.79629

```
T-TEST GROUPS=Formulation(1 2)
 /MISSING=ANALYSIS
 /VARIABLES=Release
 /CRITERIA=CI(.95).
```

T-Test 3h

Notes

Output Created		12-JUL-2014 21:34:24
Comments		
Input	Active Dataset	DataSet2
	Filter	<none>
	Weight	<none>
	Split File	<none>
	N of Rows in Working Data File	6
Missing Value Handling	Definition of Missing	User defined missing values are treated as missing.
	Cases Used	Statistics for each analysis are based on the cases with no missing or out-of-range data for any variable in the analysis.
Syntax		T-TEST GROUPS=Formulation(1 2)<br>/MISSING=ANALYSIS<br>/VARIABLES=Release<br>/CRITERIA=CI(.95).
Resources	Processor Time	00:00:00.00
	Elapsed Time	00:00:00.00

Group Statistics

	Formulation	N	Mean	Std. Deviation	Std. Error Mean
Release	BSA ph5	3	26.0000	2.00000	1.15470
	BSA ph7.4	3	20.0333	3.72872	2.15278

Independent Samples Test

		Levene's Test for Equality of Variances		t-test for Equality of Means	
		F	Sig.	t	df
Release	Equal variances assumed	1.086	.356	2.442	4
	Equal variances not assumed			2.442	3.063

Independent Samples Test

		t-test for Equality of Means			
		Sig. (2-tailed)	Mean Difference	Std. Error Difference	95% Confidence ... Lower
Release	Equal variances assumed	.071	5.96667	2.44290	-.81592
	Equal variances not assumed	.091	5.96667	2.44290	-1.71829

Independent Samples Test

		t-test for Equality of Means<br>95% Confidence Interval of the ...<br>Upper
Release	Equal variances assumed	12.74925
	Equal variances not assumed	13.65163

```
T-TEST GROUPS=Formulation(1 2)
 /MISSING=ANALYSIS
 /VARIABLES=Release
 /CRITERIA=CI(.95).
```

T-Test 4h

Notes

Output Created		12-JUL-2014 21:36:30
Comments		
Input	Active Dataset	DataSet2
	Filter	<none>
	Weight	<none>
	Split File	<none>
	N of Rows in Working Data File	6
Missing Value Handling	Definition of Missing	User defined missing values are treated as missing.
	Cases Used	Statistics for each analysis are based on the cases with no missing or out-of-range data for any variable in the analysis.
Syntax		T-TEST GROUPS=Formulation(1 2) /MISSING=ANALYSIS /VARIABLES=Release /CRITERIA=CI(.95).
Resources	Processor Time	00:00:00.00
	Elapsed Time	00:00:00.01

Group Statistics

	Formulation	N	Mean	Std. Deviation	Std. Error Mean
Release	BSA ph5	3	31.0000	1.00000	.57735
	BSA ph7.4	3	26.0000	4.58258	2.64575

Independent Samples Test

		Levene's Test for Equality of Variances		t-test for Equality of Means	
		F	Sig.	t	df
Release	Equal variances assumed	4.571	.099	1.846	4
	Equal variances not assumed			1.846	2.190

Independent Samples Test

		t-test for Equality of Means			
		Sig. (2-tailed)	Mean Difference	Std. Error Difference	95% Confidence ... Lower
Release	Equal variances assumed	.139	5.00000	2.70801	-2.51865
	Equal variances not assumed	.195	5.00000	2.70801	-5.73439

Independent Samples Test

		t-test for Equality of Means 95% Confidence Interval of the ... Upper
Release	Equal variances assumed	12.51865
	Equal variances not assumed	15.73439

```
T-TEST GROUPS=Formulation(1 2)
 /MISSING=ANALYSIS
 /VARIABLES=Release
 /CRITERIA=CI(.95).
```

T-Test 24h

Notes

Output Created		12-JUL-2014 21:37:52
Comments		
Input	Active Dataset	DataSet2
	Filter	<none>
	Weight	<none>
	Split File	<none>
	N of Rows in Working Data File	6
Missing Value Handling	Definition of Missing	User defined missing values are treated as missing.
	Cases Used	Statistics for each analysis are based on the cases with no missing or out-of-range data for any variable in the analysis.
Syntax		T-TEST GROUPS=Formulation(1 2) /MISSING=ANALYSIS /VARIABLES=Release /CRITERIA=CI(.95).
Resources	Processor Time	00:00:00.02
	Elapsed Time	00:00:00.01

Group Statistics

	Formulation	N	Mean	Std. Deviation	Std. Error Mean
Release	BSA ph5	3	40.0000	3.00000	1.73205
	BSA ph7.4	3	32.0000	3.60555	2.08167

Independent Samples Test

		Levene's Test for Equality of Variances		t-test for Equality of Means	
		F	Sig.	t	df
Release	Equal variances assumed	.250	.643	2.954	4
	Equal variances not assumed			2.954	3.872

Independent Samples Test

		t-test for Equality of Means			
					95% Confidence ...
		Sig. (2-tailed)	Mean Difference	Std. Error Difference	Lower
Release	Equal variances assumed	.042	8.00000	2.70801	.48135
	Equal variances not assumed	.044	8.00000	2.70801	.38231

Independent Samples Test

		t-test for Equality of Means
		95% Confidence Interval of the ...
		Upper
Release	Equal variances assumed	15.51865
	Equal variances not assumed	15.61769

```
T-TEST GROUPS=Formulation(1 2)
 /MISSING=ANALYSIS
 /VARIABLES=Release
 /CRITERIA=CI(.95).
```

T-Test 48h

Notes

Output Created		12-JUL-2014 21:38:57
Comments		
Input	Active Dataset	DataSet2
	Filter	<none>
	Weight	<none>
	Split File	<none>
	N of Rows in Working Data File	6
Missing Value Handling	Definition of Missing	User defined missing values are treated as missing.
	Cases Used	Statistics for each analysis are based on the cases with no missing or out-of-range data for any variable in the analysis.
Syntax		T-TEST GROUPS=Formulation(1 2) /MISSING=ANALYSIS /VARIABLES=Release /CRITERIA=CI(.95).
Resources	Processor Time	00:00:00.00
	Elapsed Time	00:00:00.00

Group Statistics

	Formulation	N	Mean	Std. Deviation	Std. Error Mean
Release	BSA ph5	3	46.0667	3.57258	2.06263
	BSA ph7.4	3	37.0000	2.00000	1.15470

Independent Samples Test

		Levene's Test for Equality of Variances		t-test for Equality of Means	
		F	Sig.	t	df
Release	Equal variances assumed	1.854	.245	3.836	4
	Equal variances not assumed			3.836	3.141

Independent Samples Test

		t-test for Equality of Means			
		Sig. (2-tailed)	Mean Difference	Std. Error Difference	95% Confidence ... Lower
Release	Equal variances assumed	.019	9.06667	2.36385	2.50357
	Equal variances not assumed	.029	9.06667	2.36385	1.73192

Independent Samples Test

		t-test for Equality of Means 95% Confidence Interval of the ... Upper
Release	Equal variances assumed	15.62976
	Equal variances not assumed	16.40141

```
T-TEST GROUPS=Formulation(1 2)
 /MISSING=ANALYSIS
 /VARIABLES=Release
 /CRITERIA=CI(.95).
```

T-Test 96h

Notes

Output Created		12-JUL-2014 21:41:03
Comments		
Input	Active Dataset	DataSet2
	Filter	<none>
	Weight	<none>
	Split File	<none>
	N of Rows in Working Data File	6
Missing Value Handling	Definition of Missing	User defined missing values are treated as missing.
	Cases Used	Statistics for each analysis are based on the cases with no missing or out-of-range data for any variable in the analysis.
Syntax		T-TEST GROUPS=Formulation(1 2) /MISSING=ANALYSIS /VARIABLES=Release /CRITERIA=CI(.95).
Resources	Processor Time	00:00:00.00
	Elapsed Time	00:00:00.00

Group Statistics

	Formulation	N	Mean	Std. Deviation	Std. Error Mean
Release	BSA ph5	3	53.0000	4.58258	2.64575
	BSA ph7.4	3	45.0000	2.64575	1.52753

Independent Samples Test

		Levene's Test for Equality of Variances		t-test for Equality of Means	
		F	Sig.	t	df
Release	Equal variances assumed	1.000	.374	2.619	4
	Equal variances not assumed			2.619	3.200

Independent Samples Test

		t-test for Equality of Means			
		Sig. (2-tailed)	Mean Difference	Std. Error Difference	95% Confidence ... Lower
Release	Equal variances assumed	.059	8.00000	3.05505	-.48218
	Equal variances not assumed	.074	8.00000	3.05505	-1.38765

Independent Samples Test

		t-test for Equality of Means
		95% Confidence Interval of the ...
		Upper
Release	Equal variances assumed	16.48218
	Equal variances not assumed	17.38765

```
T-TEST GROUPS=Formulation(1 2)
 /MISSING=ANALYSIS
 /VARIABLES=Release
 /CRITERIA=CI(.95).
```

T-Test 120h

Notes

Output Created		12-JUL-2014 21:42:51
Comments		
Input	Active Dataset	DataSet2
	Filter	<none>
	Weight	<none>
	Split File	<none>
	N of Rows in Working Data File	6
Missing Value Handling	Definition of Missing	User defined missing values are treated as missing.
	Cases Used	Statistics for each analysis are based on the cases with no missing or out-of-range data for any variable in the analysis.
Syntax		T-TEST GROUPS=Formulation(1 2) /MISSING=ANALYSIS /VARIABLES=Release /CRITERIA=CI(.95).
Resources	Processor Time	00:00:00.00
	Elapsed Time	00:00:00.01

Group Statistics

	Formulation	N	Mean	Std. Deviation	Std. Error Mean
Release	BSA ph5	3	55.0000	3.46410	2.00000
	BSA ph7.4	3	47.0667	2.90057	1.67465

Independent Samples Test

		Levene's Test for Equality of Variances		t-test for Equality of Means	
		F	Sig.	t	df
Release	Equal variances assumed	.378	.572	3.041	4
	Equal variances not assumed			3.041	3.880

Independent Samples Test

		t-test for Equality of Means			
		Sig. (2-tailed)	Mean Difference	Std. Error Difference	95% Confidence ... Lower
Release	Equal variances assumed	.038	7.93333	2.60853	.69088
	Equal variances not assumed	.040	7.93333	2.60853	.60185

Independent Samples Test

		t-test for Equality of Means 95% Confidence Interval of the ... Upper
Release	Equal variances assumed	15.17578
	Equal variances not assumed	15.26482

```
T-TEST GROUPS=Formulation(1 2)
 /MISSING=ANALYSIS
 /VARIABLES=Release
 /CRITERIA=CI(.95).
```

T-Test 216h

Notes

Output Created		12-JUL-2014 21:44:59
Comments		
Input	Active Dataset	DataSet2
	Filter	<none>
	Weight	<none>
	Split File	<none>
	N of Rows in Working Data File	6
Missing Value Handling	Definition of Missing	User defined missing values are treated as missing.
	Cases Used	Statistics for each analysis are based on the cases with no missing or out-of-range data for any variable in the analysis.
Syntax		T-TEST GROUPS=Formulation(1 2) /MISSING=ANALYSIS /VARIABLES=Release /CRITERIA=CI(.95).
Resources	Processor Time	00:00:00.00
	Elapsed Time	00:00:00.01

Group Statistics

	Formulation	N	Mean	Std. Deviation	Std. Error Mean
Release	BSA ph5	3	62.0000	2.00000	1.15470
	BSA ph7.4	3	54.0000	2.00000	1.15470

Independent Samples Test

		Levene's Test for Equality of Variances		t-test for Equality of Means	
		F	Sig.	t	df
Release	Equal variances assumed	.000	1.000	4.899	4
	Equal variances not assumed			4.899	4.000

Independent Samples Test

		t-test for Equality of Means			
					95% Confidence ...
		Sig. (2-tailed)	Mean Difference	Std. Error Difference	Lower
Release	Equal variances assumed	.008	8.00000	1.63299	3.46608
	Equal variances not assumed	.008	8.00000	1.63299	3.46608

Independent Samples Test

		t-test for Equality of Means
		95% Confidence Interval of the ...
		Upper
Release	Equal variances assumed	12.53392
	Equal variances not assumed	12.53392
