# Supplementary material for: Nanoscale Diblock Copolymer Micelles: Characterizations and Estimation of the Effective Diffusion Coefficients of Biomolecules Release through Cylindrical Diffusion Model
Source: PLoS One. 2014 Aug 18;9(8):e105234. doi: 10.1371/journal.pone.0105234 (PMC4136833; doi:10.1371/journal.pone.0105234)
Supplement: Table S2 — Statistical analysis of siRNA release from CA-PEI micelles. (PDF) [file pone.0105234.s002.pdf]

```
DATASET ACTIVATE DataSet2.
T-TEST GROUPS=Formulation(1 2)
  /MISSING=ANALYSIS
  /VARIABLES=Release
  /CRITERIA=CI(.95).
```

T-Test 0h

Notes

Output Created		12-JUL-2014 21:57:42
Comments		
Input	Active Dataset	DataSet2
	Filter	<none>
	Weight	<none>
	Split File	<none>
	N of Rows in Working Data File	6
Missing Value Handling	Definition of Missing	User defined missing values are treated as missing.
	Cases Used	Statistics for each analysis are based on the cases with no missing or out-of-range data for any variable in the analysis.
Syntax		T-TEST GROUPS=Formulation(1 2)<br>/MISSING=ANALYSIS<br>/VARIABLES=Release<br>/CRITERIA=CI(.95).
Resources	Processor Time	00:00:00.00
	Elapsed Time	00:00:00.01

[DataSet2]

Group Statistics

	Formulation	N	Mean	Std. Deviation	Std. Error Mean
Release	siRNA ph5	3	3.0000	2.29129	1.32288
	siRNA ph7.4	3	1.5000	.87178	.50332

Independent Samples Test

		Levene's Test for Equality of Variances		t-test for Equality of Means	
		F	Sig.	t	df
Release	Equal variances assumed	2.550	.186	1.060	4
	Equal variances not assumed			1.060	2.567

Independent Samples Test

		t-test for Equality of Means			
					95% Confidence ...
		Sig. (2-tailed)	Mean Difference	Std. Error Difference	Lower
Release	Equal variances assumed	.349	1.50000	1.41539	-2.42976
	Equal variances not assumed	.379	1.50000	1.41539	-3.46608

Independent Samples Test

		t-test for Equality of Means
		95% Confidence Interval of the ...
		Upper
Release	Equal variances assumed	5.42976
	Equal variances not assumed	6.46608

```
T-TEST GROUPS=Formulation(1 2)
 /MISSING=ANALYSIS
 /VARIABLES=Release
 /CRITERIA=CI(.95).
```

T-Test 1h

Notes

Output Created		12-JUL-2014 21:59:26
Comments		
Input	Active Dataset	DataSet2
	Filter	<none>
	Weight	<none>
	Split File	<none>
	N of Rows in Working Data File	6
Missing Value Handling	Definition of Missing	User defined missing values are treated as missing.
	Cases Used	Statistics for each analysis are based on the cases with no missing or out-of-range data for any variable in the analysis.
Syntax		T-TEST GROUPS=Formulation(1 2)<br>/MISSING=ANALYSIS<br>/VARIABLES=Release<br>/CRITERIA=CI(.95).
Resources	Processor Time	00:00:00.02
	Elapsed Time	00:00:00.01

Group Statistics

	Formulation	N	Mean	Std. Deviation	Std. Error Mean
Release	siRNA ph5	3	25.0000	3.60555	2.08167
	siRNA ph7.4	3	18.0000	2.05183	1.18462

Independent Samples Test

		Levene's Test for Equality of Variances		t-test for Equality of Means	
		F	Sig.	t	df
Release	Equal variances assumed	1.336	.312	2.923	4
	Equal variances not assumed			2.923	3.172

Independent Samples Test

		t-test for Equality of Means			
		Sig. (2-tailed)	Mean Difference	Std. Error Difference	95% Confidence ... Lower
Release	Equal variances assumed	.043	7.00000	2.39513	.35004
	Equal variances not assumed	.057	7.00000	2.39513	-.39329

Independent Samples Test

		t-test for Equality of Means
		95% Confidence Interval of the ...
		Upper
Release	Equal variances assumed	13.64996
	Equal variances not assumed	14.39329

```
T-TEST GROUPS=Formulation(1 2)
 /MISSING=ANALYSIS
 /VARIABLES=Release
 /CRITERIA=CI(.95).
```

T-Test 3h

Notes

Output Created		12-JUL-2014 22:00:37
Comments		
Input	Active Dataset	DataSet2
	Filter	<none>
	Weight	<none>
	Split File	<none>
	N of Rows in Working Data File	6
Missing Value Handling	Definition of Missing	User defined missing values are treated as missing.
	Cases Used	Statistics for each analysis are based on the cases with no missing or out-of-range data for any variable in the analysis.
Syntax		T-TEST GROUPS=Formulation(1 2) /MISSING=ANALYSIS /VARIABLES=Release /CRITERIA=CI(.95).
Resources	Processor Time	00:00:00.02
	Elapsed Time	00:00:00.01

Group Statistics

	Formulation	N	Mean	Std. Deviation	Std. Error Mean
Release	siRNA ph5	3	36.0000	4.00000	2.30940
	siRNA ph7.4	3	28.0000	1.00000	.57735

Independent Samples Test

		Levene's Test for Equality of Variances		t-test for Equality of Means	
		F	Sig.	t	df
Release	Equal variances assumed	2.118	.219	3.361	4
	Equal variances not assumed			3.361	2.249

Independent Samples Test

		t-test for Equality of Means			
					95% Confidence ...
		Sig. (2-tailed)	Mean Difference	Std. Error Difference	Lower
Release	Equal variances assumed	.028	8.00000	2.38048	1.39074
	Equal variances not assumed	.066	8.00000	2.38048	-1.22876

Independent Samples Test

		t-test for Equality of Means
		95% Confidence Interval of the ...
		Upper
Release	Equal variances assumed	14.60926
	Equal variances not assumed	17.22876

```
T-TEST GROUPS=Formulation(1 2)
 /MISSING=ANALYSIS
 /VARIABLES=Release
 /CRITERIA=CI(.95).
```

T-Test 4h

Group Statistics

	Formulation	N	Mean	Std. Deviation	Std. Error Mean
Release	siRNA ph5	3	43.0000	1.00000	.57735
	siRNA ph7.4	3	36.0000	3.60555	2.08167

Notes

Output Created		12-JUL-2014 22:02:12
Comments		
Input	Active Dataset	DataSet2
	Filter	<none>
	Weight	<none>
	Split File	<none>
	N of Rows in Working Data File	6
Missing Value Handling	Definition of Missing	User defined missing values are treated as missing.
	Cases Used	Statistics for each analysis are based on the cases with no missing or out-of-range data for any variable in the analysis.
Syntax		T-TEST GROUPS=Formulation(1 2)<br>/MISSING=ANALYSIS<br>/VARIABLES=Release<br>/CRITERIA=CI(.95).
Resources	Processor Time	00:00:00.03
	Elapsed Time	00:00:00.01

Independent Samples Test

		Levene's Test for Equality of Variances		t-test for Equality of Means	
		F	Sig.	t	df
Release	Equal variances assumed	4.500	.101	3.240	4
	Equal variances not assumed			3.240	2.306

Independent Samples Test

		t-test for Equality of Means			
					95% Confidence ...
		Sig. (2-tailed)	Mean Difference	Std. Error Difference	Lower
Release	Equal variances assumed	.032	7.00000	2.16025	1.00219
	Equal variances not assumed	.069	7.00000	2.16025	-1.20770

Independent Samples Test

		t-test for Equality of Means
		95% Confidence Interval of the ...
		Upper
Release	Equal variances assumed	12.99781
	Equal variances not assumed	15.20770

```
T-TEST GROUPS=Formulation(1 2)
 /MISSING=ANALYSIS
 /VARIABLES=Release
 /CRITERIA=CI(.95).
```

T-Test 24h

Notes

Output Created		12-JUL-2014 22:03:34
Comments		
Input	Active Dataset	DataSet2
	Filter	<none>
	Weight	<none>
	Split File	<none>
	N of Rows in Working Data File	6
Missing Value Handling	Definition of Missing	User defined missing values are treated as missing.
	Cases Used	Statistics for each analysis are based on the cases with no missing or out-of-range data for any variable in the analysis.
Syntax		T-TEST GROUPS=Formulation(1 2) /MISSING=ANALYSIS /VARIABLES=Release /CRITERIA=CI(.95).
Resources	Processor Time	00:00:00.00
	Elapsed Time	00:00:00.01

Group Statistics

	Formulation	N	Mean	Std. Deviation	Std. Error Mean
Release	siRNA ph5	3	52.0000	2.64575	1.52753
	siRNA ph7.4	3	45.0333	3.22542	1.86220

Independent Samples Test

		Levene's Test for Equality of Variances		t-test for Equality of Means	
		F	Sig.	t	df
Release	Equal variances assumed	.071	.804	2.892	4
	Equal variances not assumed			2.892	3.853

Independent Samples Test

		t-test for Equality of Means			
					95% Confidence ...
		Sig. (2-tailed)	Mean Difference	Std. Error Difference	Lower
Release	Equal variances assumed	.044	6.96667	2.40855	.27946
	Equal variances not assumed	.047	6.96667	2.40855	.17739

Independent Samples Test

		t-test for Equality of Means
		95% Confidence Interval of the ...
		Upper
Release	Equal variances assumed	13.65387
	Equal variances not assumed	13.75595

```
T-TEST GROUPS=Formulation(1 2)
 /MISSING=ANALYSIS
 /VARIABLES=Release
 /CRITERIA=CI(.95).
```

Notes

Output Created		12-JUL-2014 22:04:39
Comments		
Input	Active Dataset	DataSet2
	Filter	<none>
	Weight	<none>
	Split File	<none>
	N of Rows in Working Data File	6
Missing Value Handling	Definition of Missing	User defined missing values are treated as missing.
	Cases Used	Statistics for each analysis are based on the cases with no missing or out-of-range data for any variable in the analysis.
Syntax		T-TEST GROUPS=Formulation(1 2)<br>/MISSING=ANALYSIS<br>/VARIABLES=Release<br>/CRITERIA=CI(.95).
Resources	Processor Time	00:00:00.02
	Elapsed Time	00:00:00.01

Group Statistics

	Formulation	N	Mean	Std. Deviation	Std. Error Mean
Release	siRNA ph5	3	58.0000	4.35890	2.51661
	siRNA ph7.4	3	50.0000	2.69072	1.55349

Independent Samples Test

		Levene's Test for Equality of Variances		t-test for Equality of Means	
		F	Sig.	t	df
Release	Equal variances assumed	1.493	.289	2.705	4
	Equal variances not assumed			2.705	3.331

Independent Samples Test

		t-test for Equality of Means			
		Sig. (2-tailed)	Mean Difference	Std. Error Difference	95% Confidence ... Lower
Release	Equal variances assumed	.054	8.00000	2.95748	-.21127
	Equal variances not assumed	.066	8.00000	2.95748	-.90447

Independent Samples Test

		t-test for Equality of Means
		95% Confidence Interval of the ... Upper
Release	Equal variances assumed	16.21127
	Equal variances not assumed	16.90447

```
T-TEST GROUPS=Formulation(1 2)
 /MISSING=ANALYSIS
 /VARIABLES=Release
 /CRITERIA=CI(.95).
```

T-Test 91h

Notes

Output Created		12-JUL-2014 22:05:49
Comments		
Input	Active Dataset	DataSet2
	Filter	<none>
	Weight	<none>
	Split File	<none>
	N of Rows in Working Data File	6
Missing Value Handling	Definition of Missing	User defined missing values are treated as missing.
	Cases Used	Statistics for each analysis are based on the cases with no missing or out-of-range data for any variable in the analysis.
Syntax		T-TEST GROUPS=Formulation(1 2) /MISSING=ANALYSIS /VARIABLES=Release /CRITERIA=CI(.95).
Resources	Processor Time	00:00:00.00
	Elapsed Time	00:00:00.01

Group Statistics

	Formulation	N	Mean	Std. Deviation	Std. Error Mean
Release	siRNA ph5	3	65.0000	5.00000	2.88675
	siRNA ph7.4	3	56.0000	4.58258	2.64575

Independent Samples Test

		Levene's Test for Equality of Variances		t-test for Equality of Means	
		F	Sig.	t	df
Release	Equal variances assumed	.000	1.000	2.298	4
	Equal variances not assumed			2.298	3.970

Independent Samples Test

		t-test for Equality of Means				
		Sig. (2-tailed)	Mean Difference	Std. Error Difference	95% Confidence ... Lower	95% Confidence Interval of the ... Upper
Release	Equal variances assumed	.083	9.00000	3.91578	-1.87195	19.87195
	Equal variances not assumed	.084	9.00000	3.91578	-1.90445	19.90445

```
T-TEST GROUPS=Formulation(1 2)
 /MISSING=ANALYSIS
 /VARIABLES=Release
 /CRITERIA=CI(.95).
```

T-Test 115 h

Notes

Output Created		12-JUL-2014 22:07:21
Comments		
Input	Active Dataset	DataSet2
	Filter	<none>
	Weight	<none>
	Split File	<none>
	N of Rows in Working Data File	6
Missing Value Handling	Definition of Missing	User defined missing values are treated as missing.
	Cases Used	Statistics for each analysis are based on the cases with no missing or out-of-range data for any variable in the analysis.
Syntax		T-TEST GROUPS=Formulation(1 2)<br>/MISSING=ANALYSIS<br>/VARIABLES=Release<br>/CRITERIA=CI(.95).
Resources	Processor Time	00:00:00.00
	Elapsed Time	00:00:00.00

Group Statistics

	Formulation	N	Mean	Std. Deviation	Std. Error Mean
Release	siRNA ph5	3	68.0000	2.64575	1.52753
	siRNA ph7.4	3	58.0000	4.85695	2.80416

Independent Samples Test

		Levene's Test for Equality of Variances		t-test for Equality of Means	
		F	Sig.	t	df
Release	Equal variances assumed	.673	.458	3.132	4
	Equal variances not assumed			3.132	3.091

Independent Samples Test

		t-test for Equality of Means			
		Sig. (2-tailed)	Mean Difference	Std. Error Difference	95% Confidence ... Lower
Release	Equal variances assumed	.035	10.00000	3.19322	1.13419
	Equal variances not assumed	.050	10.00000	3.19322	.00472

Independent Samples Test

		t-test for Equality of Means
		95% Confidence Interval of the ...
		Upper
Release	Equal variances assumed	18.86581
	Equal variances not assumed	19.99528

```
T-TEST GROUPS=Formulation(1 2)
 /MISSING=ANALYSIS
 /VARIABLES=Release
 /CRITERIA=CI(.95).
```

T-Test 215 h

Notes

Output Created		12-JUL-2014 22:08:34
Comments		
Input	Active Dataset	DataSet2
	Filter	<none>
	Weight	<none>
	Split File	<none>
	N of Rows in Working Data File	6
Missing Value Handling	Definition of Missing	User defined missing values are treated as missing.
	Cases Used	Statistics for each analysis are based on the cases with no missing or out-of-range data for any variable in the analysis.
Syntax		T-TEST GROUPS=Formulation(1 2) /MISSING=ANALYSIS /VARIABLES=Release /CRITERIA=CI(.95).
Resources	Processor Time	00:00:00.02
	Elapsed Time	00:00:00.01

Group Statistics

	Formulation	N	Mean	Std. Deviation	Std. Error Mean
Release	siRNA ph5	3	75.0000	1.73494	1.00167
	siRNA ph7.4	3	63.0000	2.00000	1.15470

Independent Samples Test

		Levene's Test for Equality of Variances		t-test for Equality of Means	
		F	Sig.	t	df
Release	Equal variances assumed	.000	1.000	7.850	4
	Equal variances not assumed			7.850	3.922

Independent Samples Test

		t-test for Equality of Means			
					95% Confidence ...
		Sig. (2-tailed)	Mean Difference	Std. Error Difference	Lower
Release	Equal variances assumed	.001	12.00000	1.52862	7.75588
	Equal variances not assumed	.002	12.00000	1.52862	7.72229

Independent Samples Test

		t-test for Equality of Means
		95% Confidence Interval of the ...
		Upper
Release	Equal variances assumed	16.24412
	Equal variances not assumed	16.27771
