# Supplementary material for: Nanoscale Diblock Copolymer Micelles: Characterizations and Estimation of the Effective Diffusion Coefficients of Biomolecules Release through Cylindrical Diffusion Model
Source: PLoS One. 2014 Aug 18;9(8):e105234. doi: 10.1371/journal.pone.0105234 (PMC4136833; doi:10.1371/journal.pone.0105234)
Supplement: Table S4 — Statistical analysis of the effect of BSA- and siRNA-loaded CA-PEI micelles on the cell viability of DLD-1 cells. (PDF) [file pone.0105234.s004.pdf]

```
ONEWAY T1 BY F
  /MISSING ANALYSIS
  /POSTHOC=TUKEY ALPHA(0.05).
```

Oneway

Notes

Output Created		08-SEP-2013 19:20:57
Comments		
Input	Active Dataset	DataSet1
	Filter	<none>
	Weight	<none>
	Split File	<none>
	N of Rows in Working Data File	30
Missing Value Handling	Definition of Missing	User-defined missing values are treated as missing.
	Cases Used	Statistics for each analysis are based on cases with no missing data for any variable in the analysis.
Syntax		ONEWAY T1 BY F /MISSING ANALYSIS /POSTHOC=TUKEY ALPHA(0.05).
Resources	Processor Time	00:00:00.05
	Elapsed Time	00:00:00.03

```
[DataSet1]
```

ANOVA

T1

	Sum of Squares	df	Mean Square	F	Sig.
Between Groups	200.300	9	22.256	1.932	.106
Within Groups	230.367	20	11.518		
Total	430.667	29			

Post Hoc Tests

Multiple Comparisons

Dependent Variable: T1

Tukey HSD

(I) F	(J) F	Mean Difference (I-J)	Std. Error	Sig.	95% Confidence Interval	
					Lower Bound	Upper Bound
UNTREAT	BLANK RNA	5.00000	2.77108	.726	-4.8127	14.8127
	BLANK MIC	.00000	2.77108	1.000	-9.8127	9.8127
	SIRNA1:1	4.66667	2.77108	.792	-5.1460	14.4794
	sIRNA1:3	8.00000	2.77108	.174	-1.8127	17.8127
	sIRNA3:1	1.00000	2.77108	1.000	-8.8127	10.8127
	BSA1:1	1.00000	2.77108	1.000	-8.8127	10.8127
	BSA1:3	3.00000	2.77108	.981	-6.8127	12.8127
	BSA3:1	1.00000	2.77108	1.000	-8.8127	10.8127
	BLANK BSA	.00000	2.77108	1.000	-9.8127	9.8127
BLANK RNA	UNTREAT	-5.00000	2.77108	.726	-14.8127	4.8127
	BLANK MIC	-5.00000	2.77108	.726	-14.8127	4.8127
	SIRNA1:1	-.33333	2.77108	1.000	-10.1460	9.4794
	sIRNA1:3	3.00000	2.77108	.981	-6.8127	12.8127
	sIRNA3:1	-4.00000	2.77108	.899	-13.8127	5.8127
	BSA1:1	-4.00000	2.77108	.899	-13.8127	5.8127
	BSA1:3	-2.00000	2.77108	.999	-11.8127	7.8127
	BSA3:1	-4.00000	2.77108	.899	-13.8127	5.8127
	BLANK BSA	-5.00000	2.77108	.726	-14.8127	4.8127
BLANK MIC	UNTREAT	.00000	2.77108	1.000	-9.8127	9.8127
	BLANK RNA	5.00000	2.77108	.726	-4.8127	14.8127
	SIRNA1:1	4.66667	2.77108	.792	-5.1460	14.4794
	sIRNA1:3	8.00000	2.77108	.174	-1.8127	17.8127
	sIRNA3:1	1.00000	2.77108	1.000	-8.8127	10.8127
	BSA1:1	1.00000	2.77108	1.000	-8.8127	10.8127
	BSA1:3	3.00000	2.77108	.981	-6.8127	12.8127
	BSA3:1	1.00000	2.77108	1.000	-8.8127	10.8127
	BLANK BSA	.00000	2.77108	1.000	-9.8127	9.8127
SIRNA1:1	UNTREAT	-4.66667	2.77108	.792	-14.4794	5.1460
	BLANK RNA	.33333	2.77108	1.000	-9.4794	10.1460
	BLANK MIC	-4.66667	2.77108	.792	-14.4794	5.1460
	sIRNA1:3	3.33333	2.77108	.963	-6.4794	13.1460
	sIRNA3:1	-3.66667	2.77108	.936	-13.4794	6.1460
	BSA1:1	-3.66667	2.77108	.936	-13.4794	6.1460
	BSA1:3	-1.66667	2.77108	1.000	-11.4794	8.1460
	BSA3:1	-3.66667	2.77108	.936	-13.4794	6.1460
	BLANK BSA	-4.66667	2.77108	.792	-14.4794	5.1460
sIRNA1:3	UNTREAT	-8.00000	2.77108	.174	-17.8127	1.8127
	BLANK RNA	-3.00000	2.77108	.981	-12.8127	6.8127
	BLANK MIC	-8.00000	2.77108	.174	-17.8127	1.8127

Multiple Comparisons

Dependent Variable: T1

Tukey HSD

(I) F	(J) F	Mean Difference (I-J)	Std. Error	Sig.	95% Confidence Interval	
					Lower Bound	Upper Bound
	SIRNA1:1	-3.33333	2.77108	.963	-13.1460	6.4794
	sIRNA3:1	-7.00000	2.77108	.312	-16.8127	2.8127
	BSA1:1	-7.00000	2.77108	.312	-16.8127	2.8127
	BSA1:3	-5.00000	2.77108	.726	-14.8127	4.8127
	BSA3:1	-7.00000	2.77108	.312	-16.8127	2.8127
	BLANK BSA	-8.00000	2.77108	.174	-17.8127	1.8127
sIRNA3:1	UNTREAT	-1.00000	2.77108	1.000	-10.8127	8.8127
	BLANK RNA	4.00000	2.77108	.899	-5.8127	13.8127
	BLANK MIC	-1.00000	2.77108	1.000	-10.8127	8.8127
	SIRNA1:1	3.66667	2.77108	.936	-6.1460	13.4794
	sIRNA1:3	7.00000	2.77108	.312	-2.8127	16.8127
	BSA1:1	.00000	2.77108	1.000	-9.8127	9.8127
	BSA1:3	2.00000	2.77108	.999	-7.8127	11.8127
	BSA3:1	.00000	2.77108	1.000	-9.8127	9.8127
	BLANK BSA	-1.00000	2.77108	1.000	-10.8127	8.8127
BSA1:1	UNTREAT	-1.00000	2.77108	1.000	-10.8127	8.8127
	BLANK RNA	4.00000	2.77108	.899	-5.8127	13.8127
	BLANK MIC	-1.00000	2.77108	1.000	-10.8127	8.8127
	SIRNA1:1	3.66667	2.77108	.936	-6.1460	13.4794
	sIRNA1:3	7.00000	2.77108	.312	-2.8127	16.8127
	sIRNA3:1	.00000	2.77108	1.000	-9.8127	9.8127
	BSA1:3	2.00000	2.77108	.999	-7.8127	11.8127
	BSA3:1	.00000	2.77108	1.000	-9.8127	9.8127
	BLANK BSA	-1.00000	2.77108	1.000	-10.8127	8.8127
BSA1:3	UNTREAT	-3.00000	2.77108	.981	-12.8127	6.8127
	BLANK RNA	2.00000	2.77108	.999	-7.8127	11.8127
	BLANK MIC	-3.00000	2.77108	.981	-12.8127	6.8127
	SIRNA1:1	1.66667	2.77108	1.000	-8.1460	11.4794
	sIRNA1:3	5.00000	2.77108	.726	-4.8127	14.8127
	sIRNA3:1	-2.00000	2.77108	.999	-11.8127	7.8127
	BSA1:1	-2.00000	2.77108	.999	-11.8127	7.8127
	BSA3:1	-2.00000	2.77108	.999	-11.8127	7.8127
	BLANK BSA	-3.00000	2.77108	.981	-12.8127	6.8127
BSA3:1	UNTREAT	-1.00000	2.77108	1.000	-10.8127	8.8127
	BLANK RNA	4.00000	2.77108	.899	-5.8127	13.8127
	BLANK MIC	-1.00000	2.77108	1.000	-10.8127	8.8127
	SIRNA1:1	3.66667	2.77108	.936	-6.1460	13.4794
	sIRNA1:3	7.00000	2.77108	.312	-2.8127	16.8127
	sIRNA3:1	.00000	2.77108	1.000	-9.8127	9.8127

Multiple Comparisons

Dependent Variable: T1

Tukey HSD

(I) F	(J) F	Mean Difference (I-J)	Std. Error	Sig.	95% Confidence Interval	
					Lower Bound	Upper Bound
	BSA1:1	.00000	2.77108	1.000	-9.8127	9.8127
	BSA1:3	2.00000	2.77108	.999	-7.8127	11.8127
	BLANK BSA	-1.00000	2.77108	1.000	-10.8127	8.8127
BLANK BSA	UNTREAT	.00000	2.77108	1.000	-9.8127	9.8127
	BLANK RNA	5.00000	2.77108	.726	-4.8127	14.8127
	BLANK MIC	.00000	2.77108	1.000	-9.8127	9.8127
	SIRNA1:1	4.66667	2.77108	.792	-5.1460	14.4794
	sIRNA1:3	8.00000	2.77108	.174	-1.8127	17.8127
	sIRNA3:1	1.00000	2.77108	1.000	-8.8127	10.8127
	BSA1:1	1.00000	2.77108	1.000	-8.8127	10.8127
	BSA1:3	3.00000	2.77108	.981	-6.8127	12.8127
	BSA3:1	1.00000	2.77108	1.000	-8.8127	10.8127

Homogeneous Subsets

T1

Tukey HSD[a]

F	N	Subset for alpha = 0.05
		1
sIRNA1:3	3	92.0000
BLANK RNA	3	95.0000
SIRNA1:1	3	95.3333
BSA1:3	3	97.0000
sIRNA3:1	3	99.0000
BSA1:1	3	99.0000
BSA3:1	3	99.0000
UNTREAT	3	100.0000
BLANK MIC	3	100.0000
BLANK BSA	3	100.0000
Sig.		.174

Means for groups in homogeneous subsets are displayed.

a. Uses Harmonic Mean Sample Size = 3.000.
