# Supplementary material for: Nanoscale Diblock Copolymer Micelles: Characterizations and Estimation of the Effective Diffusion Coefficients of Biomolecules Release through Cylindrical Diffusion Model
Source: PLoS One. 2014 Aug 18;9(8):e105234. doi: 10.1371/journal.pone.0105234 (PMC4136833; doi:10.1371/journal.pone.0105234)
Supplement: Table S5 — Statistical analysis of the effect of BSA- and siRNA-loaded CA-PEI micelles on the cell viability of V79 cells. (PDF) [file pone.0105234.s005.pdf]

```
ONEWAY V79 BY F
  /MISSING ANALYSIS
  /POSTHOC=TUKEY ALPHA(0.05).
```

Oneway

Notes

Output Created		08-SEP-2013 19:19:32
Comments		
Input	Active Dataset	DataSet1
	Filter	<none>
	Weight	<none>
	Split File	<none>
	N of Rows in Working Data File	30
Missing Value Handling	Definition of Missing	User-defined missing values are treated as missing.
	Cases Used	Statistics for each analysis are based on cases with no missing data for any variable in the analysis.
Syntax		ONEWAY V79 BY F /MISSING ANALYSIS /POSTHOC=TUKEY ALPHA(0.05).
Resources	Processor Time	00:00:00.03
	Elapsed Time	00:00:00.02

```
[DataSet1]
```

ANOVA

V79

	Sum of Squares	df	Mean Square	F	Sig.
Between Groups	572.700	9	63.633	5.259	.001
Within Groups	242.000	20	12.100		
Total	814.700	29			

Post Hoc Tests

Multiple Comparisons

Dependent Variable: V79

Tukey HSD

(I) F	(J) F	Mean Difference (I-J)	Std. Error	Sig.	95% Confidence Interval	
					Lower Bound	Upper Bound
UNTREAT	BLANK RNA	.00000	2.84019	1.000	-10.0574	10.0574
	BLANK MIC	.00000	2.84019	1.000	-10.0574	10.0574
	SIRNA1:1	5.00000	2.84019	.751	-5.0574	15.0574
	sIRNA1:3	15.00000*	2.84019	.001	4.9426	25.0574
	sIRNA3:1	4.00000	2.84019	.911	-6.0574	14.0574
	BSA1:1	2.00000	2.84019	.999	-8.0574	12.0574
	BSA1:3	4.00000	2.84019	.911	-6.0574	14.0574
	BSA3:1	1.00000	2.84019	1.000	-9.0574	11.0574
	BLANK BSA	.00000	2.84019	1.000	-10.0574	10.0574
BLANK RNA	UNTREAT	.00000	2.84019	1.000	-10.0574	10.0574
	BLANK MIC	.00000	2.84019	1.000	-10.0574	10.0574
	SIRNA1:1	5.00000	2.84019	.751	-5.0574	15.0574
	sIRNA1:3	15.00000*	2.84019	.001	4.9426	25.0574
	sIRNA3:1	4.00000	2.84019	.911	-6.0574	14.0574
	BSA1:1	2.00000	2.84019	.999	-8.0574	12.0574
	BSA1:3	4.00000	2.84019	.911	-6.0574	14.0574
	BSA3:1	1.00000	2.84019	1.000	-9.0574	11.0574
	BLANK BSA	.00000	2.84019	1.000	-10.0574	10.0574
BLANK MIC	UNTREAT	.00000	2.84019	1.000	-10.0574	10.0574
	BLANK RNA	.00000	2.84019	1.000	-10.0574	10.0574
	SIRNA1:1	5.00000	2.84019	.751	-5.0574	15.0574
	sIRNA1:3	15.00000*	2.84019	.001	4.9426	25.0574
	sIRNA3:1	4.00000	2.84019	.911	-6.0574	14.0574
	BSA1:1	2.00000	2.84019	.999	-8.0574	12.0574
	BSA1:3	4.00000	2.84019	.911	-6.0574	14.0574
	BSA3:1	1.00000	2.84019	1.000	-9.0574	11.0574
	BLANK BSA	.00000	2.84019	1.000	-10.0574	10.0574
SIRNA1:1	UNTREAT	-5.00000	2.84019	.751	-15.0574	5.0574
	BLANK RNA	-5.00000	2.84019	.751	-15.0574	5.0574
	BLANK MIC	-5.00000	2.84019	.751	-15.0574	5.0574
	sIRNA1:3	10.00000	2.84019	.052	-.0574	20.0574
	sIRNA3:1	-1.00000	2.84019	1.000	-11.0574	9.0574
	BSA1:1	-3.00000	2.84019	.984	-13.0574	7.0574
	BSA1:3	-1.00000	2.84019	1.000	-11.0574	9.0574
	BSA3:1	-4.00000	2.84019	.911	-14.0574	6.0574
	BLANK BSA	-5.00000	2.84019	.751	-15.0574	5.0574
sIRNA1:3	UNTREAT	-15.00000*	2.84019	.001	-25.0574	-4.9426
	BLANK RNA	-15.00000*	2.84019	.001	-25.0574	-4.9426
	BLANK MIC	-15.00000*	2.84019	.001	-25.0574	-4.9426

Multiple Comparisons

Dependent Variable: V79

Tukey HSD

(I) F	(J) F	Mean Difference (I-J)	Std. Error	Sig.	95% Confidence Interval Lower Bound	95% Confidence Interval Upper Bound
	SIRNA1:1	-10.00000	2.84019	.052	-20.0574	.0574
	sIRNA3:1	-11.00000*	2.84019	.025	-21.0574	-.9426
	BSA1:1	-13.00000*	2.84019	.006	-23.0574	-2.9426
	BSA1:3	-11.00000*	2.84019	.025	-21.0574	-.9426
	BSA3:1	-14.00000*	2.84019	.003	-24.0574	-3.9426
	BLANK BSA	-15.00000*	2.84019	.001	-25.0574	-4.9426
sIRNA3:1	UNTREAT	-4.00000	2.84019	.911	-14.0574	6.0574
	BLANK RNA	-4.00000	2.84019	.911	-14.0574	6.0574
	BLANK MIC	-4.00000	2.84019	.911	-14.0574	6.0574
	SIRNA1:1	1.00000	2.84019	1.000	-9.0574	11.0574
	sIRNA1:3	11.00000*	2.84019	.025	.9426	21.0574
	BSA1:1	-2.00000	2.84019	.999	-12.0574	8.0574
	BSA1:3	.00000	2.84019	1.000	-10.0574	10.0574
	BSA3:1	-3.00000	2.84019	.984	-13.0574	7.0574
	BLANK BSA	-4.00000	2.84019	.911	-14.0574	6.0574
BSA1:1	UNTREAT	-2.00000	2.84019	.999	-12.0574	8.0574
	BLANK RNA	-2.00000	2.84019	.999	-12.0574	8.0574
	BLANK MIC	-2.00000	2.84019	.999	-12.0574	8.0574
	SIRNA1:1	3.00000	2.84019	.984	-7.0574	13.0574
	sIRNA1:3	13.00000*	2.84019	.006	2.9426	23.0574
	sIRNA3:1	2.00000	2.84019	.999	-8.0574	12.0574
	BSA1:3	2.00000	2.84019	.999	-8.0574	12.0574
	BSA3:1	-1.00000	2.84019	1.000	-11.0574	9.0574
	BLANK BSA	-2.00000	2.84019	.999	-12.0574	8.0574
BSA1:3	UNTREAT	-4.00000	2.84019	.911	-14.0574	6.0574
	BLANK RNA	-4.00000	2.84019	.911	-14.0574	6.0574
	BLANK MIC	-4.00000	2.84019	.911	-14.0574	6.0574
	SIRNA1:1	1.00000	2.84019	1.000	-9.0574	11.0574
	sIRNA1:3	11.00000*	2.84019	.025	.9426	21.0574
	sIRNA3:1	.00000	2.84019	1.000	-10.0574	10.0574
	BSA1:1	-2.00000	2.84019	.999	-12.0574	8.0574
	BSA3:1	-3.00000	2.84019	.984	-13.0574	7.0574
	BLANK BSA	-4.00000	2.84019	.911	-14.0574	6.0574
BSA3:1	UNTREAT	-1.00000	2.84019	1.000	-11.0574	9.0574
	BLANK RNA	-1.00000	2.84019	1.000	-11.0574	9.0574
	BLANK MIC	-1.00000	2.84019	1.000	-11.0574	9.0574
	SIRNA1:1	4.00000	2.84019	.911	-6.0574	14.0574
	sIRNA1:3	14.00000*	2.84019	.003	3.9426	24.0574
	sIRNA3:1	3.00000	2.84019	.984	-7.0574	13.0574

Multiple Comparisons

Dependent Variable: V79

Tukey HSD

(I) F	(J) F	Mean Difference (I-J)	Std. Error	Sig.	95% Confidence Interval	
					Lower Bound	Upper Bound
BLANK BSA	BSA1:1	1.00000	2.84019	1.000	-9.0574	11.0574
	BSA1:3	3.00000	2.84019	.984	-7.0574	13.0574
	BLANK BSA	-1.00000	2.84019	1.000	-11.0574	9.0574
	UNTREAT	.00000	2.84019	1.000	-10.0574	10.0574
	BLANK RNA	.00000	2.84019	1.000	-10.0574	10.0574
	BLANK MIC	.00000	2.84019	1.000	-10.0574	10.0574
	SIRNA1:1	5.00000	2.84019	.751	-5.0574	15.0574
	sIRNA1:3	15.00000*	2.84019	.001	4.9426	25.0574
	sIRNA3:1	4.00000	2.84019	.911	-6.0574	14.0574
	BSA1:1	2.00000	2.84019	.999	-8.0574	12.0574
	BSA1:3	4.00000	2.84019	.911	-6.0574	14.0574
	BSA3:1	1.00000	2.84019	1.000	-9.0574	11.0574

*. The mean difference is significant at the 0.05 level.

Homogeneous Subsets

V79

Tukey HSD[a]

F	N	Subset for alpha = 0.05	
		1	2
sIRNA1:3	3	85.0000	
SIRNA1:1	3	95.0000	95.0000
sIRNA3:1	3		96.0000
BSA1:3	3		96.0000
BSA1:1	3		98.0000
BSA3:1	3		99.0000
UNTREAT	3		100.0000
BLANK RNA	3		100.0000
BLANK MIC	3		100.0000
BLANK BSA	3		100.0000
Sig.		.052	.751

Means for groups in homogeneous subsets are displayed.

a. Uses Harmonic Mean Sample Size = 3.000.
