# Supplementary material for: Nanoscale Diblock Copolymer Micelles: Characterizations and Estimation of the Effective Diffusion Coefficients of Biomolecules Release through Cylindrical Diffusion Model
Source: PLoS One. 2014 Aug 18;9(8):e105234. doi: 10.1371/journal.pone.0105234 (PMC4136833; doi:10.1371/journal.pone.0105234)
Supplement: Table S6 — Statistical analysis of the BSA and siRNA loading efficiency of CA-PEI micelles. (PDF) [file pone.0105234.s006.pdf]

```
ONEWAY ee BY f
  /MISSING ANALYSIS
  /POSTHOC=TUKEY ALPHA(0.05).
```

Oneway

Notes

Output Created		08-SEP-2013 18:52:26
Comments		
Input	Active Dataset	DataSet0
	Filter	<none>
	Weight	<none>
	Split File	<none>
	N of Rows in Working Data File	27
Missing Value Handling	Definition of Missing	User-defined missing values are treated as missing.
	Cases Used	Statistics for each analysis are based on cases with no missing data for any variable in the analysis.
Syntax		ONEWAY ee BY f /MISSING ANALYSIS /POSTHOC=TUKEY ALPHA(0.05).
Resources	Processor Time	00:00:00.00
	Elapsed Time	00:00:00.02

```
[DataSet0]
```

ANOVA

ee

	Sum of Squares	df	Mean Square	F	Sig.
Between Groups	2765.340	5	553.068	452.414	.000
Within Groups	14.670	12	1.222		
Total	2780.010	17			

Post Hoc Tests

Multiple Comparisons

Dependent Variable: ee

Tukey HSD

(I) f	(J) f	Mean Difference (I-J)	Std. Error	Sig.	95% Confidence Interval	
					Lower Bound	Upper Bound
SIRNA11	SIRNA13	-2.00000	.90277	.298	-5.0323	1.0323
	SIRNA31	1.30000	.90277	.705	-1.7323	4.3323
	BSA11	24.70000*	.90277	.000	21.6677	27.7323
	BSA13	21.00000*	.90277	.000	17.9677	24.0323
	BSA31	27.00000*	.90277	.000	23.9677	30.0323
SIRNA13	SIRNA11	2.00000	.90277	.298	-1.0323	5.0323
	SIRNA31	3.30000*	.90277	.030	.2677	6.3323
	BSA11	26.70000*	.90277	.000	23.6677	29.7323
	BSA13	23.00000*	.90277	.000	19.9677	26.0323
	BSA31	29.00000*	.90277	.000	25.9677	32.0323
SIRNA31	SIRNA11	-1.30000	.90277	.705	-4.3323	1.7323
	SIRNA13	-3.30000*	.90277	.030	-6.3323	-.2677
	BSA11	23.40000*	.90277	.000	20.3677	26.4323
	BSA13	19.70000*	.90277	.000	16.6677	22.7323
	BSA31	25.70000*	.90277	.000	22.6677	28.7323
BSA11	SIRNA11	-24.70000*	.90277	.000	-27.7323	-21.6677
	SIRNA13	-26.70000*	.90277	.000	-29.7323	-23.6677
	SIRNA31	-23.40000*	.90277	.000	-26.4323	-20.3677
	BSA13	-3.70000*	.90277	.014	-6.7323	-.6677
	BSA31	2.30000	.90277	.185	-.7323	5.3323
BSA13	SIRNA11	-21.00000*	.90277	.000	-24.0323	-17.9677
	SIRNA13	-23.00000*	.90277	.000	-26.0323	-19.9677
	SIRNA31	-19.70000*	.90277	.000	-22.7323	-16.6677
	BSA11	3.70000*	.90277	.014	.6677	6.7323
	BSA31	6.00000*	.90277	.000	2.9677	9.0323
BSA31	SIRNA11	-27.00000*	.90277	.000	-30.0323	-23.9677
	SIRNA13	-29.00000*	.90277	.000	-32.0323	-25.9677
	SIRNA31	-25.70000*	.90277	.000	-28.7323	-22.6677
	BSA11	-2.30000	.90277	.185	-5.3323	.7323
	BSA13	-6.00000*	.90277	.000	-9.0323	-2.9677

*. The mean difference is significant at the 0.05 level.

Homogeneous Subsets

ee

Tukey HSD[a]

f	N	Subset for alpha = 0.05			
		1	2	3	4
BSA31	3	68.0000			
BSA11	3	70.3000			
BSA13	3		74.0000		
SIRNA31	3			93.7000	
SIRNA11	3			95.0000	95.0000
SIRNA13	3				97.0000
Sig.		.185	1.000	.705	.298

Means for groups in homogeneous subsets are displayed.

a. Uses Harmonic Mean Sample Size = 3.000.
