# Supplementary material for: Nanoscale Diblock Copolymer Micelles: Characterizations and Estimation of the Effective Diffusion Coefficients of Biomolecules Release through Cylindrical Diffusion Model
Source: PLoS One. 2014 Aug 18;9(8):e105234. doi: 10.1371/journal.pone.0105234 (PMC4136833; doi:10.1371/journal.pone.0105234)
Supplement: Table S7 — Statistical analysis of the zeta potential of blank, BSA- and siRNA-loaded CA-PEI micelles. (PDF) [file pone.0105234.s007.pdf]

```
ONEWAY zeta BY f
  /MISSING ANALYSIS
  /POSTHOC=TUKEY ALPHA(0.05).
```

Oneway

Notes

Output Created		08-SEP-2013 18:44:26
Comments		
Input	Active Dataset	DataSet0
	Filter	<none>
	Weight	<none>
	Split File	<none>
	N of Rows in Working Data File	27
Missing Value Handling	Definition of Missing	User-defined missing values are treated as missing.
	Cases Used	Statistics for each analysis are based on cases with no missing data for any variable in the analysis.
Syntax		ONEWAY zeta BY f /MISSING ANALYSIS /POSTHOC=TUKEY ALPHA(0.05).
Resources	Processor Time	00:00:00.03
	Elapsed Time	00:00:00.02

[DataSet0]

ANOVA

zeta

	Sum of Squares	df	Mean Square	F	Sig.
Between Groups	443.507	8	55.438	50.045	.000
Within Groups	19.940	18	1.108		
Total	463.447	26			

Post Hoc Tests

Multiple Comparisons

Dependent Variable: zeta

Tukey HSD

(I) f	(J) f	Mean Difference (I-J)	Std. Error	Sig.	95% Confidence Interval Lower Bound	Upper Bound
B11	B13	-6.50000*	.85937	.000	-9.5111	-3.4889
	B31	3.00000	.85937	.051	-.0111	6.0111
	SIRNA11	5.40000*	.85937	.000	2.3889	8.4111
	SIRNA13	9.00000*	.85937	.000	5.9889	12.0111
	SIRNA31	4.40000*	.85937	.002	1.3889	7.4111
	BSA11	2.10000	.85937	.319	-.9111	5.1111
	BSA13	.50000	.85937	1.000	-2.5111	3.5111
	BSA31	3.90000*	.85937	.006	.8889	6.9111
B13	B11	6.50000*	.85937	.000	3.4889	9.5111
	B31	9.50000*	.85937	.000	6.4889	12.5111
	SIRNA11	11.90000*	.85937	.000	8.8889	14.9111
	SIRNA13	15.50000*	.85937	.000	12.4889	18.5111
	SIRNA31	10.90000*	.85937	.000	7.8889	13.9111
	BSA11	8.60000*	.85937	.000	5.5889	11.6111
	BSA13	7.00000*	.85937	.000	3.9889	10.0111
	BSA31	10.40000*	.85937	.000	7.3889	13.4111
B31	B11	-3.00000	.85937	.051	-6.0111	.0111
	B13	-9.50000*	.85937	.000	-12.5111	-6.4889
	SIRNA11	2.40000	.85937	.184	-.6111	5.4111
	SIRNA13	6.00000*	.85937	.000	2.9889	9.0111
	SIRNA31	1.40000	.85937	.778	-1.6111	4.4111
	BSA11	-.90000	.85937	.975	-3.9111	2.1111
	BSA13	-2.50000	.85937	.151	-5.5111	.5111
	BSA31	.90000	.85937	.975	-2.1111	3.9111
SIRNA11	B11	-5.40000*	.85937	.000	-8.4111	-2.3889
	B13	-11.90000*	.85937	.000	-14.9111	-8.8889
	B31	-2.40000	.85937	.184	-5.4111	.6111
	SIRNA13	3.60000*	.85937	.013	.5889	6.6111
	SIRNA31	-1.00000	.85937	.955	-4.0111	2.0111
	BSA11	-3.30000*	.85937	.026	-6.3111	-.2889
	BSA13	-4.90000*	.85937	.001	-7.9111	-1.8889
	BSA31	-1.50000	.85937	.714	-4.5111	1.5111
SIRNA13	B11	-9.00000*	.85937	.000	-12.0111	-5.9889
	B13	-15.50000*	.85937	.000	-18.5111	-12.4889

Multiple Comparisons

Dependent Variable: zeta

Tukey HSD

(I) f	(J) f	Mean Difference (I-J)	Std. Error	Sig.	95% Confidence Interval	
					Lower Bound	Upper Bound
	B31	-6.00000*	.85937	.000	-9.0111	-2.9889
	SIRNA11	-3.60000*	.85937	.013	-6.6111	-.5889
	SIRNA31	-4.60000*	.85937	.001	-7.6111	-1.5889
	BSA11	-6.90000*	.85937	.000	-9.9111	-3.8889
	BSA13	-8.50000*	.85937	.000	-11.5111	-5.4889
	BSA31	-5.10000*	.85937	.000	-8.1111	-2.0889
SIRNA31	B11	-4.40000*	.85937	.002	-7.4111	-1.3889
	B13	-10.90000*	.85937	.000	-13.9111	-7.8889
	B31	-1.40000	.85937	.778	-4.4111	1.6111
	SIRNA11	1.00000	.85937	.955	-2.0111	4.0111
	SIRNA13	4.60000*	.85937	.001	1.5889	7.6111
	BSA11	-2.30000	.85937	.223	-5.3111	.7111
	BSA13	-3.90000*	.85937	.006	-6.9111	-.8889
	BSA31	-.50000	.85937	1.000	-3.5111	2.5111
BSA11	B11	-2.10000	.85937	.319	-5.1111	.9111
	B13	-8.60000*	.85937	.000	-11.6111	-5.5889
	B31	.90000	.85937	.975	-2.1111	3.9111
	SIRNA11	3.30000*	.85937	.026	.2889	6.3111
	SIRNA13	6.90000*	.85937	.000	3.8889	9.9111
	SIRNA31	2.30000	.85937	.223	-.7111	5.3111
	BSA13	-1.60000	.85937	.645	-4.6111	1.4111
	BSA31	1.80000	.85937	.505	-1.2111	4.8111
BSA13	B11	-.50000	.85937	1.000	-3.5111	2.5111
	B13	-7.00000*	.85937	.000	-10.0111	-3.9889
	B31	2.50000	.85937	.151	-.5111	5.5111
	SIRNA11	4.90000*	.85937	.001	1.8889	7.9111
	SIRNA13	8.50000*	.85937	.000	5.4889	11.5111
	SIRNA31	3.90000*	.85937	.006	.8889	6.9111
	BSA11	1.60000	.85937	.645	-1.4111	4.6111
	BSA31	3.40000*	.85937	.020	.3889	6.4111
BSA31	B11	-3.90000*	.85937	.006	-6.9111	-.8889
	B13	-10.40000*	.85937	.000	-13.4111	-7.3889
	B31	-.90000	.85937	.975	-3.9111	2.1111
	SIRNA11	1.50000	.85937	.714	-1.5111	4.5111
	SIRNA13	5.10000*	.85937	.000	2.0889	8.1111
	SIRNA31	.50000	.85937	1.000	-2.5111	3.5111
	BSA11	-1.80000	.85937	.505	-4.8111	1.2111
	BSA13	-3.40000*	.85937	.020	-6.4111	-.3889

*. The mean difference is significant at the 0.05 level.

Homogeneous Subsets

zeta

Tukey HSD[a]

f	N	Subset for alpha = 0.05				
		1	2	3	4	5
SIRNA13	3	3.2000				
SIRNA11	3		6.8000			
SIRNA31	3		7.8000	7.8000		
BSA31	3		8.3000	8.3000		
B31	3		9.2000	9.2000	9.2000	
BSA11	3			10.1000	10.1000	
BSA13	3				11.7000	
B11	3				12.2000	
B13	3					18.7000
Sig.		1.000	.184	.223	.051	1.000

Means for groups in homogeneous subsets are displayed.

a. Uses Harmonic Mean Sample Size = 3.000.
